# Targeting granulosa cells with engineered DFO nanoparticles for the treatment of chemotherapy-induced premature ovarian failure

**DOI:** 10.7150/thno.115416

**Published:** 2025-07-04

**Authors:** Tuo Zhang, Zhiyi Sheng, Jixian Zhang, Heng Zhang, Yan Zhang, Yunpeng Du, Xiangxin Liu, Zhu Hu, Qiyuan Luo, Guoqiang Xu, Yuan Gao, Meina He, Tengxiang Chen

**Affiliations:** 1 Transformation Engineering Research Center of Chronic Disease Diagnosis and Treatment, Department of Physiology, College of Basic Medicine, Guizhou Medical University, Guiyang, Guizhou, 561113, China.; 2 Guizhou Provincial Key Laboratory of Pathogenesis & Drug Research on Common Chronic Diseases, Guizhou Medical University, Guiyang, Guizhou, 561113, China.; 3 Center for Reproductive Medicine, Shandong University, Jinan, 250012, China.

**Keywords:** premature ovarian failure, the labile iron pool, ferroptosis, ferritinophagy, engineered DFO nanoparticles

## Abstract

Premature ovarian failure (POF) is a prevalent and lethal adverse event that severely affects female cancer patients receiving chemotherapy. It is highly correlated with the collateral damage to ovarian granulosa cells caused by ferroptosis. The excessive iron accumulation in the intracellular labile iron pool (LIP) and the aberrant generation of reactive oxygen species (ROS) is the major cause of ferroptosis. No compounds with both ferroptosis inhibition functions and ovarian-targeting specificity that do not reduce systemic chemotherapeutic efficacy have been successful in treating the POF in the clinic trials. We herein in report a multifunctional FSH-mPDA@DFO nanoparticle that can prevent chemotherapy-induced DFO by targeting LIP and ROS to restrict ferroptosis. The FSH-mPDA@DFO are constructed by encapsulating deferoxamine (DFO) within mesoporous polydopamine nanoparticles conjugated with an ovarian granulosa cell-targeting peptide (FSHβ). They downregulate transferrin receptor (TFRC) expression, reduce cellular iron ion uptake, and inhibit ferritinophagy-mediated iron mobilization, thereby ameliorating LIP overload and regulating iron metabolism. More importantly, FSH-mPDA@DFO nanoparticles can specifically accumulate in the mitochondria and suppress excessive mitophagy to preserve mitochondrial integrity and attenuate iron release and ROS generation. *In vivo* experiments demonstrate that FSH-mPDA@DFO nanoparticles improve oocyte quantity and quality while restoring fertility and endocrine homeostasis in chemotherapy-induced POF mice. Collectively, FSH-mPDA@DFO nanoparticles hold a great potential for clinical prevention and treatment of POF patients.

## Introduction

Premature ovarian failure (POF) is a devastating condition characterized by the premature loss of ovarian function, leading to infertility, profound endocrine disruptions, and systemic hypoestrogenism-associated complications, including osteoporosis and cardiovascular morbidity 1. Chemotherapy-induced POF is particularly prevalent in younger women, with nearly one-third of cancer patients under the age of 40 developing POF as a consequence of chemotherapy, particularly due to the cytotoxic effects on ovarian follicles. Currently, available treatments, such as hormone replacement therapy, fail to restore ovarian function or fertility, leaving a critical gap in care for reproductive-age women. Ovarian tissue cryopreservation offers a potential fertility-preserving approach, but it is limited by the risk of malignant cell reintroduction following transplantation 2. This highlights the urgent need for targeted therapies capable of restoring ovarian function and fertility, particularly for chemotherapy-induced POF.

Ovarian granulosa cells are pivotal in supporting oocyte development and follicular maturation. These cells are proliferative during the reproductive cycle, which makes them particularly susceptible to chemotherapeutic agents that induce follicular atresia 3-6. Recent studies have shown that chemotherapy-induced ovarian dysfunction is largely driven by granulosa cells ferroptosis 7-9. The LIP is an essential driver of ferroptosis, because it disturbs iron homeostasis and increases the production of ROS mediated by iron 10-11. High concentrations of iron in the LIP lead to chemical reactivity and cytotoxicity due to the ability of this iron to catalyze Fenton's reactions, which lead to ROS formation and overload with the potential to induce oxidative stress and cell damage. The LIP is also a rapidly adjustable source of Fe^2+^ and Fe^3+^. Excess free Fe^2+^ in the LIP can react with hydrogen peroxide (H_2_O_2_) to generate Fe^3+^ and hydroxyl radicals. This causes iron dyshomeostasis and LIP accumulation, subsequently induce ferroptosis 12. The intracellular LIP exists in a dynamic equilibrium that is strictly regulated to allow iron uptake, utilization, distribution, and export. In mammals, iron ions are transported into cells by transferrin receptor 1 (TFRC) or divalent metal-ion transporter 1 (DMT1). Controlling the cellular uptake of iron is one of the main ways to regulate the size of the intracellular iron pool. Lysosomes provide the source of iron to the LIP by degrading ferritin and reducing Fe^3+^ 13. Mitochondrial fragmentation and enhanced mitophagy release iron ions, providing an additional source of iron for ferroptosis and making cells more sensitive to ferroptosis 10. These two pathways are also important ways to increase the capacity of the intracellular labile iron pool. Therefore, LIP homeostasis is a particularly important driver of ferroptosis in POF ovarian granulosa cells. Specifically targeting the LIP may therefore be an effective yet rarely demonstrated strategy for protecting POF patients from ferroptosis.

However, conventional iron chelators, such as deferoxamine (DFO), face challenges due to suboptimal pharmacokinetics and nonspecific biodistribution, limiting their efficacy in ovarian protection 10. To overcome these limitations, we developed a novel FSH-mPDA@DFO nanoparticle platform. Unlike conventional iron-targeting nanoparticles, this platform combines FSHβ-targeted delivery with a polydopamine (mPDA) coating, enhancing nanoparticle stability and ROS-scavenging activity. This unique nanoplatform offers several advantages: (1) specific targeting to ovarian granulosa cells, (2) inhibition of transferrin receptor (TFRC)-mediated iron uptake and ferritinophagy in the lysosome, (3) suppression of excessive mitophagy to preserve mitochondrial integrity, attenuate release iron ions and ROS generation to prevent ferroptosis.

The targeting strategy of FSH-mPDA@DFO nanoparticles is based on the natural binding between follicle-stimulating hormone (FSH) and its receptor (FSHR), which is predominantly expressed on granulosa cells in the ovaries 14-15. The FSHβ component of our nanoparticle targets FSHR-expressing granulosa cells through the natural binding of FSH to its receptor, which does not rely on chemotherapy-induced upregulation of FSHR expression. FSH-mPDA@DFO nanoparticles consist of a DFO core coated with mPDA and functionalized with FSHβ, enabling precise targeting to granulosa cells. Upon cellular uptake, these nanoparticles suppress TFRC expression, inhibit ferritinophagy-mediated iron release from lysosomes, and reduce mitophagy to preserve mitochondrial integrity and attenuate mitochondrial Fe^2+^ accumulation and ROS generation, mitigating lipid peroxidation and inhibiting ferroptosis.

In summary, FSH-mPDA@DFO nanoparticles represent a pioneering ferroptosis-targeting strategy for treating chemotherapy-induced POF. This innovative approach addresses existing therapeutic challenges by restoring iron homeostasis and protecting ovarian function, with significant potential for advancing anti-ferroptosis therapeutics in reproductive medicine.

## Materials and Methods

### Animal

CAG-RFP/EGFP/LC3B transgenic reporter mice and *Foxl2-CreERT2* mice were generously provided by Professor Chao Wang from the College of Biological Sciences, China Agricultural University (Beijing, China). *Tfrc^fl/fl^* mice were obtained from Cyagen Technology Co., Ltd. Adult CD-1 and C57BL/6 mice were purchased from Beijing Vital River Laboratory Animal Technology Co., Ltd. All mice were maintained under controlled environmental conditions (12-hour light/dark cycle, temperature: 24-26 °C) with ad libitum access to food and water. The experimental protocols were approved by the Institutional Animal Care and Research Committee of Guizhou Medical University (Approval NO. 2200064). Tamoxifen (TAM) (T5648; Merck, Rahway, NJ, USA) was administered as previously described 16. Briefly, 6-week-old *Tfrc^fl/fl^* and *Tfrc^fl/fl^; Foxl2-CreERT2* female mice received intraperitoneal injections of 20 mg/kg TAM every other day for a total of three injections. The primers used for genotyping are detailed in the Supplementary Materials.

### Identification of mice estrous cycle

The estrous cycle was assessed following established protocols 17. Vaginal smears were collected and stained with hematoxylin and eosin. The stages of the estrous cycle (proestrus, estrus, metestrus, and diestrus) were determined by analyzing the morphology of vaginal epithelial cells. Continuous monitoring was performed over a 14-day period, and the estrous cycle data were analyzed and plotted using GraphPad Prism 8.

### Isolation of mice and human primary granulosa cells

Eight-week-old CAG-RFP/EGFP/LC3B transgenic reporter female mice were euthanized for the isolation of primary granulosa cells, following established protocols 18-19. Granulosa cells were isolated under minimal light exposure. Isolated primary granulosa cells were cultured with 20 μM cisplatin for 24 h. Prior to staining, 200 μM Mito-Tracker (C1032, Beyotime, China) was pre-warmed for 30 min, and cells were then incubated with a final concentration of 200 nM Mito-Tracker for 1 h. Cellular observations were conducted using a laser confocal microscope.

Granulosa cells from patients with diminished ovarian reserve (DOR) were isolated as previously described 20-21. Granulosa cells from women under 35 years of age with infertility due to male factors served as the control group. All experimental procedures were approved by the Ethics Committee of the Affiliated Hospital of Guizhou Medical University (Approval NO. 202463).

### Fertility test

Female mice treated with Cis, Cis + mPDA@DFO, or Cis + FSH-mPDA@DFO were continuously mated with 12-week-old fertile male mice for 10 weeks. The total number of pups per litter and cumulative fertility rate were recorded.

### Superovulation

Female mice treated with Cis, Cis + mPDA@DFO, or Cis + FSH-mPDA@DFO were intraperitoneally injected with 5 IU of pregnant mare serum gonadotropin (PMSG; B210807; Ningbo Sansheng Biological Technology, China). 44 h later, the mice received an intraperitoneal injection of 5 IU of human chorionic gonadotropin (hCG; B210808; Ningbo Sansheng Biological Technology, China). Oocytes were collected from the fallopian tubes 16 h later and subjected to immunofluorescence analysis.

### Assay of hormones and metabolites in serum

Blood samples were obtained from anesthetized mice via ocular phlebotomy and allowed to clot at room temperature for 30 min. The samples were then centrifuged at 3000 rpm for 20 min to collect the serum. The concentrations of estradiol (E_2_), follicle-stimulating hormone (FSH), and luteinizing hormone (LH) in the serum were measured using the Cobas e602 module of the Cobas 8000 total automation system (Roche). Additionally, the levels of alanine aminotransferase (ALT) and aspartate aminotransferase (AST) were determined using the Alanine Aminotransferase Assay Kit (C009-2-1, Nanjing Jiancheng Bioengineering Institute, China) and the Aspartate Aminotransferase Assay Kit (C010-2-1, Nanjing Jiancheng Bioengineering Institute, China), respectively, following the manufacturer's instructions.

### Hemolysis assay

Whole blood samples were collected from mice via retro-orbital venous plexus puncture. The blood was promptly transferred into EDTA-anticoagulated tubes and gently mixed through inversion. Primary centrifugation was performed at 3000 rpm for 20 min (4 °C) to separate plasma components. The supernatant was carefully aspirated, and the pelleted erythrocytes were washed three times with 0.9% saline through repetitive resuspension-centrifugation cycles (3000 rpm, 20 min) until optical clarity of the supernatant was achieved. A 5% erythrocyte suspension was created by reconstituting 50 μL of packed red blood cells in 950 μL of sterile saline. Serial dilutions of test compounds were prepared in 0.9% saline. Aliquots (1 mL) of the 5% erythrocyte suspension were transferred into 1.5 mL microcentrifuge tubes and centrifuged (3000 rpm, 10 min) to pellet the erythrocytes. The supernatants were discarded, and the erythrocyte pellets were resuspended in 1 mL of the test material solutions. Experimental controls included a negative control group (0.9% saline) and a positive control (deionized water). All samples were incubated statically at 37 °C for 2 h. After incubation, the samples were centrifuged at 10000 rpm for 20 min to pellet the intact erythrocytes. The hemolytic response was qualitatively assessed using digital documentation under standardized lighting conditions. Aliquots (150 μL) of the supernatant were analyzed spectrophotometrically at 540 nm. The percentage of hemolysis was calculated.

### Preparation of FSH-mPDA@DFO

Poloxamer F127 (P477925, Aladdin, China) and TMB monosulfate (T303960, Aladdin, China) were combined in a mixed solvent composed of water and ethanol at a 1:1 (v/v) ratio and stirred for 30 min to form a white emulsion. Subsequently, aqueous ammonia and dopamine hydrochloride were added, and the reaction proceeded at room temperature for 2 h. The resulting suspension was then centrifuged at 13000 rpm for 10 min to collect the precipitate, which was washed twice with water/ethanol and three times with ethanol and acetone before being redispersed to obtain mesoporous polydopamine (mPDA). Deferoxamine was dissolved in pure water and mixed with mPDA, then stirred overnight. After centrifugation at 13000 rpm for 10 min, the precipitate was collected, washed with water, and redispersed to yield mesoporous polydopamine loaded with deferoxamine (mPDA@DFO). The mPDA was then placed in a HEPES buffer (10 mM) at pH 8.5, and the FSH targeting peptide (YTRDLVYKDPARPKIQKTCTF) was added. The reaction was allowed to proceed at room temperature for 6 h. Following another centrifugation at 13000 rpm for 10 min, the precipitate was collected, washed with water, and redispersed to obtain FSH-mPDA@DFO.

### Cell culture

The KGN cells (WN-58445, WarnerBio, China) were maintained with Dulbecco's modified Eagle's medium (DMEM; Gibco) supplemented with 10% fetal bovine serum (FBS; Biological Industries) and 1% penicillin / streptomycin (P1400, Solarbio, China). All cell lines were cultured at 37 ℃, 5% CO2 and saturated humidity. The KGN cells were cultured with 20 μM cisplatin (ST1164, Beyotime, China), 10 μM Erastin (Era; HY-115594, MedChemExpress, USA), 1 μM Ferrostatin-1 (Fer-1; HY-100579, MedChemExpress, USA), 100 nM Deferoxamine mesylate salt (D9533, Sigma, USA).

### Cell counting Kit-8 analysis

Cell viability was determined by the Cell counting Kit-8 (CCK8) assay (GK1001, GLPBIO, USA) according to the manufacturer's instruction. The KGN cells were seeded into 96-well plates at a density of 1 × 10^4^ cells per well. 100 μL of CCK8 working solution was incubated for 2 h, and the absorbance was detected by 450 nm.

### 5-ethynyl-2′-deoxyuridine proliferation assay

5-ethynyl-2′-deoxyuridine (EdU) staining was performed with BeyoClic^TM^ EdU Cell Proliferation Kit (C0075S, Beyotime, China) according to the manufacturer's guidelines. The cells were incubated with 500 μL of diluted EdU working solution for 30 min. Cells were fixed with 4% paraformaldehyde for 20 min and stained with DAPI for 5 min. Images were captured with a confocal microscope (FV12-IXCOV, Olympus SpinSR10, Japan).

### Measurement of malondialdehyde

The content of malondialdehyde (MDA) in cell lysates was measured with Lipid Peroxidation MDA Assay Kit (S0131M, Beyotime, China). KGN cells (1 × 10^6^) were homogenized on ice in 0.1 mL of PBS and centrifuged at 10000 g for 10 min, collecting the supernatant. The MDA assay was performed according to the manufacturer's instructions.

### Mitochondrial membrane potential assay

The mitochondrial membrane potential in cells was detected with a JC-1 fluorescent probe (C2003S, Beyotime, China) according to the manufacturer's instructions. Briefly, the cells were cultured with 1 mL of JC-1 working solution for 20 min and rinsed cells twice with the JC-1 staining buffer. Images were captured with a confocal microscope (FV12-IXCOV, Olympus SpinSR10, Japan).

### Reactive oxygen species and mitochondrial superoxide assay

The intracellular levels of reactive oxygen species and superoxides in mitochondria were detected with Reactive Oxygen Species Assay Kit (S0033M, Beyotime, China) and Mitochondrial Superoxide Assay Kit with MitoSOX Red (S0061S, Beyotime, China) according to the manufacturer's instruction, respectively.

### Lipid peroxidation assay

The lipid peroxidation levels in live cells were detected with BODIPY 581/591 C11 (D3861, Invitrogen, USA) according to the manufacturer's instructions. Briefly, the cells were incubated with 5 μM BODIPY 581/591 C11 for 60 min. After incubation, remove the supernatant and wash the cells twice with PBS. Images were captured with a confocal microscope.

### Measurement of cellular labile iron pool

The chelate intracellular iron pool was determined by using the fluorescent probe Phen green SK (PGSK, P14313, Invitrogen, USA). The cells were incubated with 20 μM Phen green SK for 30 min. After incubation, washed with PBS and imaged with a confocal microscope.

### Immunofluorescence

The mouse ovaries were immersed in 4% paraformaldehyde for 12 to 16 h. Subsequently, they underwent dehydration through a series of graded alcohol solutions, clearing in xylene, embedding in paraffin, and sectioning at a thickness of 5 micrometers. The ovarian sections were then deparaffinized and rehydrated, followed by microwave-assisted antigen retrieval using 0.01% sodium citrate buffer for 20 min. After thorough rinsing with phosphate-buffered saline (PBS), the sections were blocked with 5% bovine serum albumin in PBS for 1 hour at room temperature. They were then incubated with primary antibodies overnight at 4 ℃. Following the primary antibody incubation, the ovarian sections were rigorously rinsed with PBS and incubated with Alexa Fluor 488- or 555-conjugated secondary antibodies for 1 hour at 37 ℃. After another thorough rinse with PBS, the sections were stained with 4',6-diamidino-2-phenylindole (DAPI) for 5 min to visualize the nuclei. Finally, the ovarian sections were mounted using anti-fade fluorescence mounting medium (C1210, Applygen, China) under coverslips for preservation and visualization. Sections were examined and photographed using Olympus OlyVIA. The primary and secondary antibodies used are listed in Supplementary Materials. Post hoc image analysis was performed with ImageJ software.

### Prussian blue iron staining

Prussian blue staining was performed with Prussian Blue and Nuclear Fast Red Staining Kit (G1428, Solarbio, China) according to the manufacturer's instructions. Briefly, the ovarian sections were dewaxed and rehydrated. Subsequently, 50-100 μL of acid potassium ferrocyanide solution was applied to each section and incubated at room temperature for 15 min. After incubation, the sections were washed with double-distilled water (ddH_2_O) for 5 min, dehydrated through a graded series of alcohols, cleared in xylene, and finally mounted with neutral resin. Sections were photographed with Olympus OlyVIA VS200.

### Western blotting analysis

The protein samples were extracted utilizing WIP Tissue and Cell Lysis Solution, which contained 1 mM phenylmethylsulfonyl fluoride (PMSF; 8553S, Cell Signaling Technologies, USA), in strict adherence to the manufacturer's guidelines. Subsequently, the protein concentration was quantitatively determined through the implementation of a BCA assay (P0012, Beyotime, China), following the manufacturer's instructions meticulously. The extracted protein samples were then fractionated on SDS-PAGE gels with a concentration gradient ranging from 10% to 15%, and were subsequently transferred onto polyvinylidene fluoride membranes (IPVH00010, Millipore, USA). The membranes were blocked with 5% skim milk for 1 hour at room temperature and incubated with the primary antibodies overnight at 4 ℃. After thorough rinsing with tris buffered saline with Tween-20 (TBST), the membranes were then incubated with the secondary antibody for 1 hour at room temperature, followed by another rigorous rinsing step with TBST. The membranes were visualized using a SuperSignal West Pico Chemiluminescent Detection System (5200, Tanon, China). The primary and secondary antibodies used are listed in Supplementary Materials.

### Real-time PCR analysis

The total RNA was extracted from ovaries and KGN cells using trizol reagent (15596018CN, Invitrogen, USA) following the manufacturer's instructions. First-strand cDNA was synthesized by Strand cDNA Synthesis SuperMix (11141ES60, Yeasen, China) for qPCR following the manufacturer's instructions. RT-PCR was performed using SYBR Select Master Mix with the Applied Biosystems 7500 Real Time PCR System (Applied Biosystems, Life Technologies, USA). The data were normalized by *β-actin*. Primers used are listed in Supplementary Materials.

### RNA-seq and LC-MS data analysis

The ovaries of mice injected with cisplatin for 14 days were collected for RNA-sequence (RNA-seq) and liquid chromatography-mass spectrometry (LC-MS), which were performed by Shanghai Personalbio Technology Co., Ltd. and the Mass Spectrometry Lab of the China Agricultural University (Beijing, China), respectively. The differentially expressed genes (DEGs) were identified between the two groups at a *P*-value < 5% and absolute log2FoldChange ≥ 1. Kyoto Encyclopedia of Genes and Genomes (KEGG) pathway enrichment analyses were performed using Metascape and Bioinformatics-Wei Sheng Xin (www.bioinformatics.com.cn/) 22. Gene Set Enrichment Analysis (GSEA) was performed with GSEA software, and gene sets were obtained from the GSEA database. The relative gene expression profiles were used as the input dataset. Enrichment was considered significant at an |NES| ≥ 1 and the nominal *P* value ≤ 0.05. Mfuzz analysis was performed with Mfuzz package on Bioconductor (DOI: 10.18129/B9.bioc.Mfuzz).

### Statistical analyses

Statistical analyses were carried out using GraphPad Prism 8. Data were expressed as the mean of at least three independent experiments. Results are given as means ± SEM. ANOVA was used to determine statistical differences in experiments with more than two treatment groups.

## Results

### Ferroptosis drives cisplatin induced POF in human and mice granulosa cells

**Figure 1:**
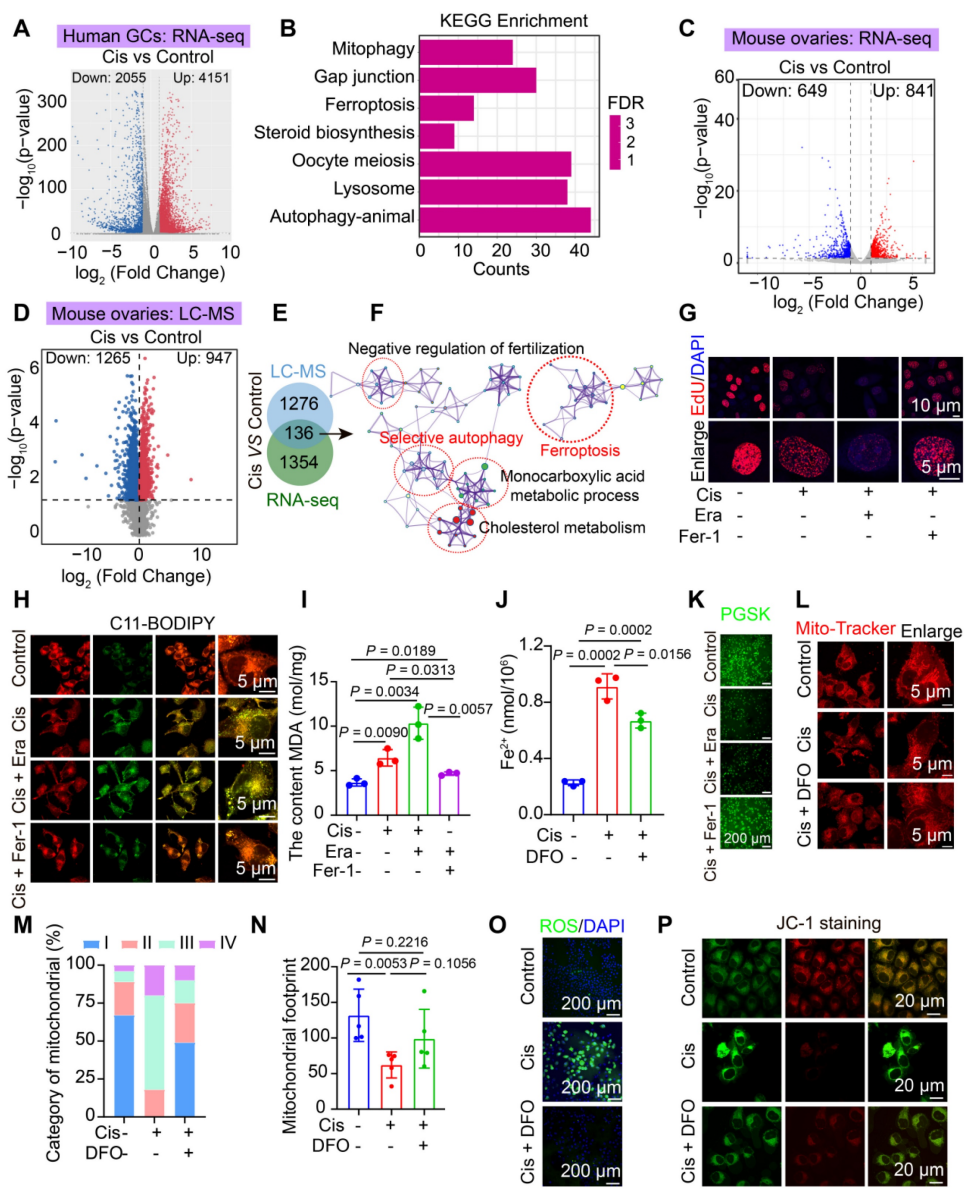
** Cisplatin chemotherapy induces ferroptosis in human and mouse granulosa cells. A-B** Volcano plot (A) and KEGG pathway enrichment analysis (B) of DEGs identified by RNA-seq in cisplatin-treated human primary granulosa cells. **C-D** Heatmap of differentially expressed genes (C) and proteins (D) identified by RNA-seq and LC-MS analysis, respectively, in mouse ovaries treated with or without cisplatin for 14 days. **E-F** Venn diagram analysis (E) and representative protein-protein interaction (PPI) networks (F) for 136 overlapping differentially expressed genes and proteins, highlighting key pathway underlying cisplatin-induced ovarian injury. **G** EdU staining and statistical analysis of KGN cells. **H** Lipid peroxidation measured using BODIPY 488/561 C11 staining. **I** Measurement of MDA content using the Lipid Peroxidation MDA Assay Kit. **J-K** Evaluation of Fe²⁺ content using Phen Gree^TM^SK. **L** Mitochondrial staining of KGN cells using Mito-Tracker Red. **M** Categorization of mitochondria within KGN cells. **N** Analysis of mitochondrial footprint. **O** Intracellular ROS levels in KGN cells measured using the Reactive Oxygen Species Assay Kit. **P** Assessment of mitochondrial membrane potential using the JC-1 assay kit.

Fertility preservation in patients undergoing chemotherapy-induced POF presents significant medical challenges. To investigate the potential signaling pathways involved in cisplatin-induced POF, we cultured human primary granulosa cells with cisplatin. We then performed RNA sequencing (RNA-seq). First, volcano plot analysis revealed a total of 2055 downregulated genes and 4151 upregulated genes in cisplatin group (Figure 1A). Subsequently, KEGG and GSEA enrichment analyses indicated that pathways associated with ferroptosis, mitophagy, gap junctions, lysosomal function, cell cycle, oocyte meiosis, and hormone regulation were significantly enriched (Figure 1B, S1A-F). These findings suggest that exposure to cisplatin induces ferroptosis and mitophagy in cisplatin cultured human primary granulosa cells.

Furthermore, we established a cisplatin-induced POF mouse model and conducted RNA-seq and LC-MS to validate the consistency between mouse and human sequencing results. Firstly, we demonstrated the successful establishment of this cisplatin-induced POF mouse model through body weight, ovary weight, ovary weight/ body weight (Figure S2A-D), the estrous cycle (Figure S2E-F) and serum sex hormone including E_2_, FSH, and LH (Figure S2G-I), HE staining and follicle counting (Figure S2J-L). Moreover, we found that the expression of senescence-associated secretory phenotype related genes, including *p16*, *p21*, *Mmp2*, *Il-6*, and *Tgf-β*, was upregulated in the cisplatin group (Figure S2M). Sirius red and Masson staining demonstrated increased ovarian interstitial fibrosis in the cisplatin-treated ovaries (Figure S2N-P), consistent with ovarian aging accompanied by increased interstitial fibrosis 23. Collectively, these data confirm the successful establishment of a cisplatin-induced POF mouse model.

Subsequently, RNA-seq and LC-MS analyses were performed based on the successfully established POF mouse model (Figure 1C-D). The RNA-seq data revealed 649 downregulated genes and 841 upregulated genes (Figure 1C), while the LC-MS data identified 1265 downregulated proteins and 947 upregulated proteins (Figure 1D). KEGG and GSEA analyses of the RNA-seq and LC-MS data demonstrated significant enrichment of several signaling pathways, including ferroptosis, autophagy, steroid hormone biosynthesis, fatty acid elongation and oxidative phosphorylation (Figure S1G-K). Additionally, KEGG and PPI analyses of the 136 overlapping genes between RNA-seq and LC-MS also found ferroptosis and autophagy were significantly enriched (Figure 1E-F). Collectively, these findings suggest that ferroptosis and mitophagy were enriched in cisplatin-induced POF mouse ovaries.

Furthermore, to confirm that cisplatin induces POF through the activation of ferroptosis in granulosa cells, we assessed key hallmarks of ferroptosis in both cisplatin induced POF cell model and mice model. First, cellular viability demonstrated a concentration- and time-dependent decrease in cell viability after cisplatin-treated KGN granulosa cells by CCK8 and EdU assays (Figure 1G, S3A). This reduction in viability was exacerbated by Era (a ferroptosis agonist) and rescued by Fer-1 (a ferroptosis inhibitor) or deferoxamine (an iron chelator) (Figure 1G, S3A-E). The core molecules involved in the regulation of the ferroptosis pathway were activated, which was evidenced by decreased expression of the antioxidant proteins GPX4 and SLC7A11, coupled with upregulation of iron metabolism regulators HMOX1, CP, PCBP1, FTL and TFRC in cisplatin treated KGN cells and mice model (Figure S3F-N). In cisplatin treated KGN cells model, lipid peroxidation levels increased, as indicated by C11-BODIPY staining and malondialdehyde (MDA) content measurement (Figure 1H-I). Furthermore, iron ion deposition was visualized using PGSK probes (Figure 1J-K). Next, Mito-Tracker was used to detect the mitochondria, the results showed mitochondria were damaged, which characterized by the disappearance of mitochondrial cristae, degenerative mitochondrial footprints, reduced mitochondrial length and aspect ratio, and an increased number of mitochondria exhibiting higher damage levels, were assessed using transmission electron microscopy and Mito-Tracker staining (Figure 1L-N, S4A-D). Elevated ROS and mitochondria ROS levels were detected using the ROS Assay Kit and Mito-SOX Red (Figure 1O, S4E-F), alongside a reduction in mitochondrial membrane potential, as indicated by JC-1 staining (Figure 1P, S4G). Notably, the addition of DFO effectively reversed these effects (Figure 1J-P, S4G). Our *in vitro* studies demonstrated that while Fer-1 partially rescued cisplatin-induced KGN cell damage (viability restored to ~ 70% of controls), DFO achieved near-complete restoration (Figure S3B-E). DFO was ultimately chosen for subsequent experiments. DFO administration increased ovarian weight, reduced follicle atresia and interstitial fibrosis, and enhanced granulosa cell proliferation compared to that observed in cisplatin-induced premature ovarian failure (Figure S5A-D). Collectively, these findings suggest that ferroptosis acts as a driving force in cisplatin-induced POF and represents a potential therapeutic target.

### Engineering and characterization of FSH-mPDA@DFO nanoparticles

**Figure 2:**
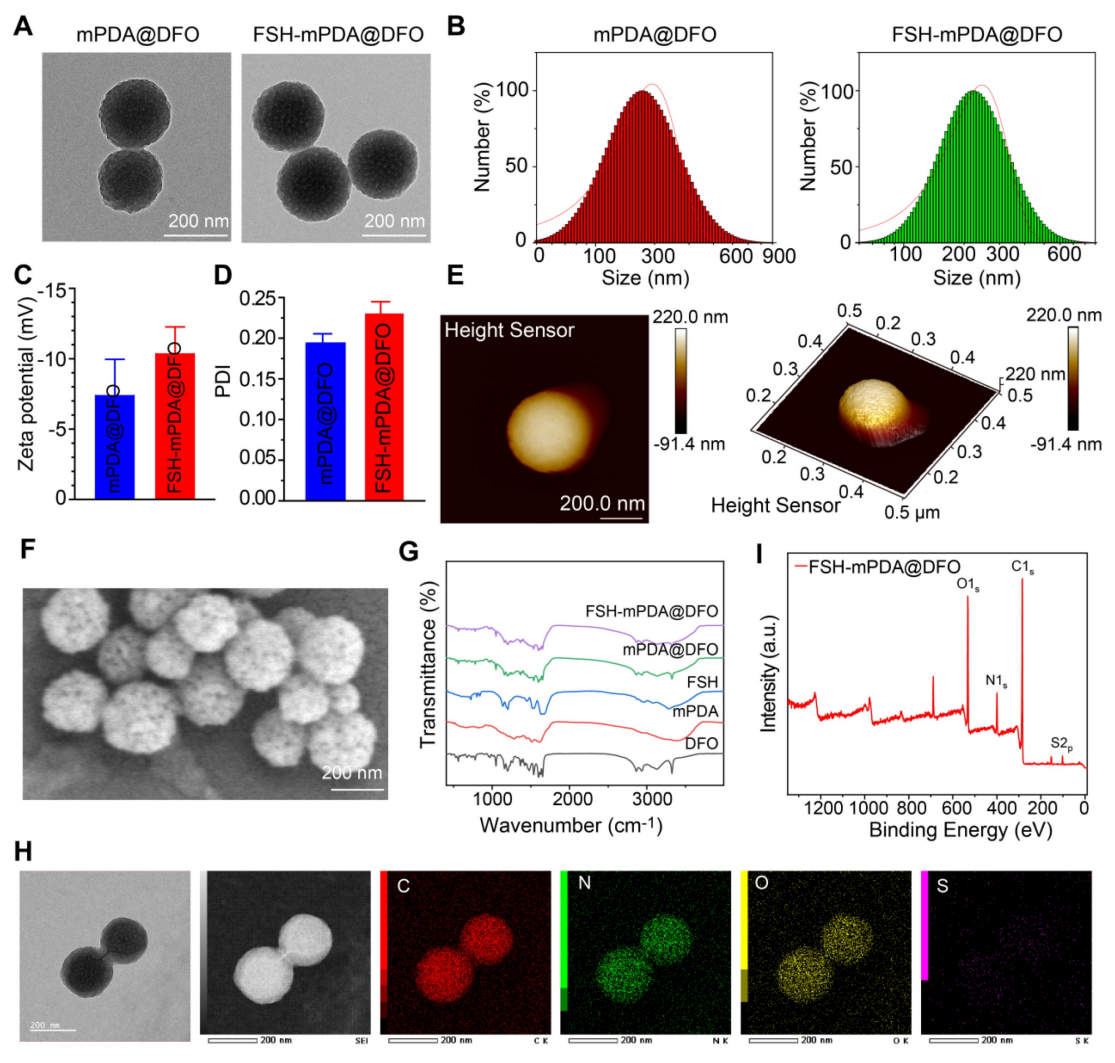
** Characterization of FSH-mPDA@DFO. A** Transmission electron microscopy images of mPDA@DFO and FSH-mPDA@DFO. **B** Particle size analysis diagram for mPDA@DFO and FSH-mPDA@DFO. **C-D** Analysis of zeta potentials and polydispersity index. **E-F** Atomic force microscopy and scanning electron microscopy images of mPDA@DFO and FSH-mPDA@DFO. **G** Fourier transform infrared spectra of various materials. **H** Scanning transmission electron microscopy image of FSH-mPDA@DFO with elemental mapping for carbon (C), nitrogen (N), oxygen (O), and sulfur (S). **I** The X-ray photoelectron spectroscopy analysis of FSH-mPDA@DFO.

FSH-mPDA@DFO nanoparticles were synthesized by first preparing mesoporous polydopamine nanoparticles (mPDA), followed by loading DFO into the mPDA pores through π-π stacking interactions, yielding mPDA@DFO nanoparticles as previously described 24. Transmission electron microscopy (TEM) confirmed the spherical morphology of mPDA@DFO nanoparticles, with an average particle size of approximately 200 nm (Figure 2A, S6A). Dynamic light scattering (DLS) analysis indicated a hydrodynamic diameter of 226.59 ± 9.97 nm (Figure 2B), and zeta potential measurements at pH 7.0 demonstrated a surface charge of - 10.4 ± 1.85 mV (Figure 2C). mPDA-based materials exhibit intrinsic chemical reactivity due to the presence of catechol and maleimide (MAL) groups, enabling surface modifications via Michael addition with thiol-containing compounds 25-26. Leveraging this property, the surface of mPDA@DFO nanoparticles was functionalized with a cysteine residue at the N-terminus of the ovary-targeting peptide FSHβ33-53. Follicle-stimulating hormone (FSH), a glycoprotein hormone composed of α and β chains, binds to the FSH receptor (FSHR), a G protein-coupled receptor. Specific FSHR-binding domains, including amino acids 33-53 of the FSHβ chain, exhibit high binding affinity 14-15. FSHβ33-53, with its superior binding affinity, was selected as a targeting ligand to direct nanoparticle delivery to ovarian tissues 27.

As previously reported, FSHβ33-53 was conjugated to mPDA@DFO nanoparticles by incubating the peptide with the nanoparticles in a pH 8.5 buffer 28. The morphology and size of FSH-mPDA@DFO nanoparticles were characterized by scanning electron microscopy (SEM), transmission electron microscope (TEM), and atomic force microscopy (AFM). TEM and SEM images revealed a spherical morphology with a mean diameter of 223 nm (Figure 2A-F), while AFM measurements indicated a thickness of approximately 220 nm (Figure 2E), confirming the successful preparation of uniformly sized nanoparticles. DLS analysis further demonstrated a hydrodynamic diameter of 248.98 ± 37.65 nm at pH 7.0 (Figure 2B), and a zeta potential of - 11.89 ± 4.07 mV (Figure 2C), which is favorable for cellular uptake and nanoparticle dispersion. The polydispersity index (PDI) of FSH-mPDA@DFO nanoparticles was 0.23 ± 0.015, indicating a narrow size distribution suitable for biomedical applications (Figure 2D).

Fourier-transform infrared spectroscopy (FT-IR) analysis revealed an absorption band at 1455 cm^-1^, which corresponds to the bending vibration of CH2 in DFO 29. This confirms the successful loading of DFO into mPDA (Figure 2G). Energy-dispersive X-ray spectroscopy (STEM-EDS) mapping confirmed homogeneous distribution of sulfur (from FSHβ33-53 cysteine residues) colocalized with carbon/nitrogen (mPDA backbone), validating spatially controlled surface functionalization (Figure 2H). Additionally, X-ray photoelectron spectroscopy (XPS) detected distinct sulfur signals, further validating the successful conjugation of FSH to the nanoparticles (Figure 2I). Collectively, these results confirm the successful synthesis and characterization of FSH-mPDA@DFO nanoparticles.

### Cellular uptake of FSH-mPDA@DFO nanoparticles

**Figure 3:**
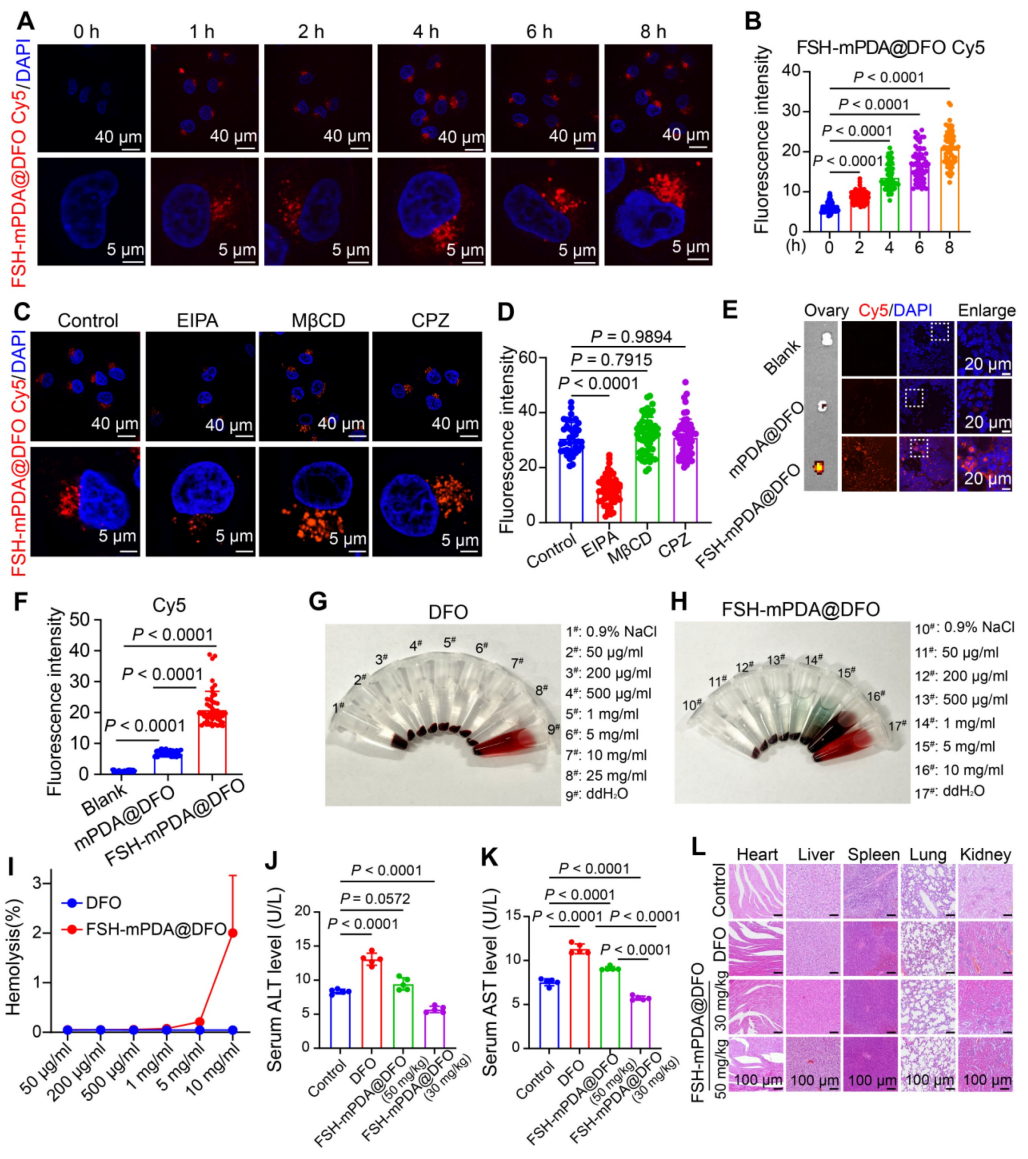
** Engineered FSH-mPDA@DFO nanoparticles exhibits favorable biosafety. A-B** Cellular uptake of FSH-mPDA@DFO in KGN cells from 0 h to 8 h, illustrated through fluorescence imaging (A) and quantitative analysis (B) (n = 50). **C-D** The channels of cellular uptake of FSH-mPDA@DFO, shown via fluorescence imaging (C) and quantitative analysis (D) (n = 50). **E-F** *Ex vivo* biodistribution analysis of CD-1 mice showing fluorescence imaging of ovarian tissues at 60 min post intravenous administration of saline, mPDA@DFO-Cy5 or FSH-mPDA@DFO-Cy5 (E). IF localization of Cy5 in ovarian granulosa cells using frozen tissue sections and quantitative analysis of fluorescence intensity, demonstrating specific targeting efficiency of FSH-mPDA@DFO (F). **G-I** Hemocompatibility assessment of DFO and FSH-mPDA@DFO through hemolysis assay, including macroscopic observation (G, H) and quantitative analysis (I). **J-K** Hepatic function evaluation through serum ALT and AST level measurement in treated mice (n = 5). **L** Histopathological examination of kidney, lung, spleen, liver, and heart tissues by HE staining.

Cellular uptake is a critical step in achieving therapeutic effects. To investigate the cellular uptake of FSH-mPDA@DFO nanoparticles in granulosa cells, we constructed Cy5-labeled FSH-mPDA@DFO nanoparticles. FSH-mPDA@DFO-Cy5 nanoparticles exhibited continuous release in PBS (pH = 7.4) over 50 h, with the most rapid cumulative release occurring between 0 and 8 h (Figure S6B). After 1 h, the FSH-mPDA@DFO-Cy5 nanoparticles were distributed throughout the cytoplasm of KGN cells, with fluorescence intensity gradually increasing from 1 to 8 h (Figure 3A-B). This indicates time-dependent cellular uptake of FSH-mPDA@DFO nanoparticles. We examined the cellular uptake pathways of FSH@DFO using the following inhibitors: methyl-β-cyclodextrin (MβCD), chlorpromazine (CPZ), and 5-(N-ethyl-N-isopropyl)-amiloride (EIPA). MβCD, CPZ, and EIPA inhibit the delivery system via lipid raft-, clathrin-, and macropinosome-mediated pathways, respectively 30. The fluorescence intensity of Cy5 in cells remained unchanged following the addition of MβCD and CPZ. However, a significant decrease in fluorescence intensity was observed with the addition of EIPA (Figure 3C-D), indicating that FSH@DFO primarily enters cells through macropinosome-mediated endocytosis. These results suggest that FSH-mPDA@DFO nanoparticles are predominantly absorbed via the macropinocytosis pathway.

### The FSH-mPDA@DFO nanoparticles display satisfactory biocompatibility *in vitro* and *in vivo*

The biological effects of nanoparticles are critical for their applications in biomedicine *in vivo*. To investigate the targeting efficiency of FSH-mPDA@DFO nanoparticles to ovarian granulosa cells and assess their biosafety, we conducted *in vivo* imaging using a small animal imaging system. Mice were intravenously administered via the tail vein with saline (blank control), Cy5-labeled mPDA@DFO (non-targeted), and Cy5-labeled FSH-mPDA@DFO (targeted), respectively. *In vivo* imaging was performed at 5, 10, 15, 20, 30, 40, and 60 minutes post-injection. The results demonstrated no detectable red fluorescence signal in the blank control group (Figure S7A). In the mPDA@DFO group, red fluorescence appeared throughout the body within 5 min (1/3 mice) and in all mice by 10 min, consistent with nanoparticles entering circulation. The signal peaked at 15 min but declined significantly by 30 min, disappearing by 60 min (except faint residual spots), aligning with DFO's short blood half-life (20-30 min). This indicates non-specific distribution followed by hepatic/renal clearance, confirmed by *ex vivo* imaging showing residual liver/kidney signals (Figure 3E, Figure S7A-D). In the FSH-mPDA@DFO group, signal at 5-15 min was notably weaker than mPDA@DFO due to receptor-ligand binding kinetics slowing systemic dispersion. Signal peaked at 20 min and persisted in the abdominal region (ovarian site) at 30-60 min. By 60 min, fluorescence was localized predominantly to ovaries (Figure 3E/F), with no signal in non-target organs (*ex vivo data*). The prolonged ovarian retention occurs because FSH binds to FSH receptors on ovarian granulosa cells (Figure 3E, Figure S7A-B). This was validated by ovarian frozen sections showing Cy5 signal co-localized with granulosa cells (Figure 3E-F). In summary, the targeted formulation exhibits delayed but sustained ovarian accumulation due to active FSH-receptor binding, whereas non-targeted nanoparticles undergo rapid systemic distribution and clearance. The delayed whole-body signal intensity peak and prolonged abdominal retention observed in the FSH-mPDA@DFO group reflect altered pharmacokinetics driven by FSH-mediated active targeting. This ligand functionalization enables ovarian granulosa cell-specific delivery, this confirms the successful development of an ovary-specific DFO delivery system.

To assess the biosafety of FSH-mPDA@DFO nanoparticles *in vivo*, we first conducted hemolysis tests to evaluate blood compatibility. The hemolysis rate after treatment with ddH_2_O reached 100%, whereas the hemolysis rates after treatment with 0.9% NaCl, various concentrations of DFO and FSH-mPDA@DFO nanoparticles were all below 5% (Figure 3G-I). This suggests that FSH-mPDA@DFO exhibits no hemolytic activity and possesses favorable blood compatibility. Iron chelators like DFO may also induce hepatotoxicity under prolonged or high-dose exposure due to iron depletion interfering with metabolic pathways 31-32. Monitoring body weight over 14 days showed a gradual decrease in the cisplatin group compared to the control group. Notably, the body weights of mice in the mPDA@DFO and FSH-mPDA@DFO groups remained comparable to those in the control group (Figure S8). Additionally, analysis revealed increased levels of ALT and AST, biochemical indicators of liver damage, in the serum of mice in the DFO group. Importantly, the levels of ALT and AST in the 50 mg/kg FSH-mPDA@DFO group were comparable to those in the control group, indicating its suitability for further research. In contrast, ALT and AST levels in the serum of mice in the 30 mg/kg FSH-mPDA@DFO group were lower than those in the control group (Figure 3J-K). This suggests that FSH-mPDA@DFO, which targets ovarian granulosa cells, may mitigate the hepatic toxicity associated with DFO. Histological examination using HE staining revealed that FSH-mPDA@DFO did not induce structural damage to the heart, liver, spleen, lungs, or kidneys (Figure 3L). Collectively, these findings indicate that FSH-mPDA@DFO demonstrates better biosafety compared to DFO in mice.

### FSH-mPDA@DFO nanoparticles reduce iron accumulation and protect against cisplatin-induced POF

**Figure 4:**
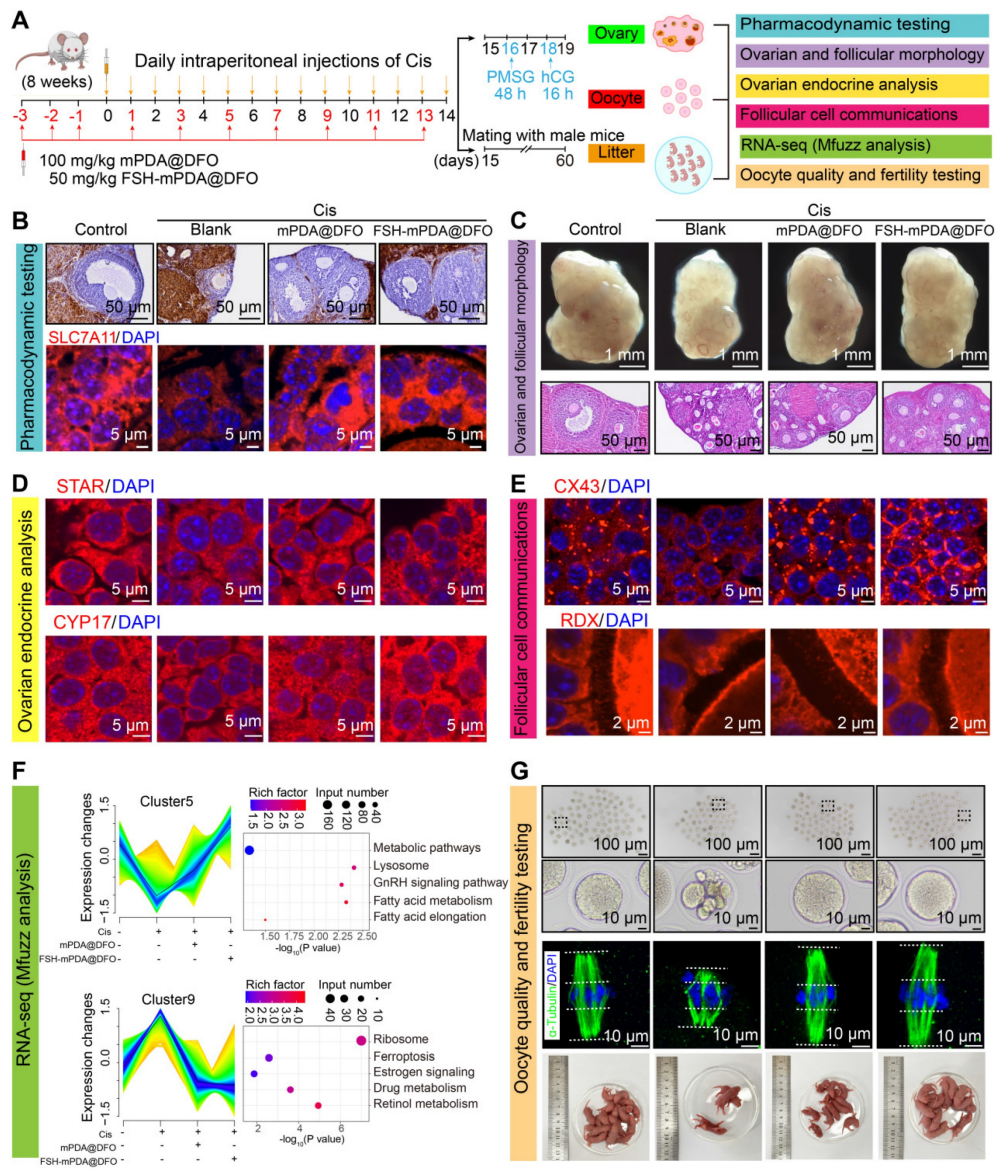
** FSH-mPDA@DFO protects against chemotherapy-induced POF *in vivo*. A** Schematic representation of the treatment schedule and therapy assessments for cisplatin-induced POF in mice. **B** Efficiency assay of FSH-mPDA@DFO for inhibiting ferroptosis *in vivo*. Prussian Blue staining and IF staining of SLC17A1 demonstrated that FSH-mPDA@DFO effectively inhibited ferroptosis in the ovaries. **C** Representative photographs of the ovaries and HE sections of the ovaries from each treatment group. **D** Endocrine analysis of ovaries. IF staining of STAR and CYP17 was performed for the visualization of ovarian endocrine function following treatment with cisplatin, mPDA@DFO, and FSH-mPDA@DFO. **E** Examination of follicular communication between oocytes and granulosa cells in ovaries after treatment with cisplatin, mPDA@DFO, and FSH-mPDA@DFO. **F** Mfuzz and KEGG analyses of RNA-seq data following treatment with cisplatin, cisplatin + mPDA@DFO, and cisplatin + FSH-mPDA@DFO; assessment of oocyte quality and fertility. **G** Oocyte quality assessed through morphological observation and α-Tubulin IF staining after treatment with cisplatin, mPDA@DFO, and FSH-mPDA@DFO. Female mice were housed with wild-type males to evaluate fertility after treatment with cisplatin, cisplatin + mPDA@DFO, and cisplatin + FSH-mPDA@DFO.

To investigate the therapeutic potential of FSH-mPDA@DFO nanoparticles in cisplatin-induced POF, we assessed three major aspects: the ovary, oocytes, and litter size (Figure 4A). The iron-chelation activities of FSH-mPDA@DFO nanoparticles were evaluated in the POF mouse model. Prussian blue staining revealed excessive iron accumulation in the cisplatin group, which was significantly diminished following treatment with mPDA@DFO and FSH-mPDA@DFO nanoparticles (Figure 4B). Moreover, IF staining demonstrated a decrease in SLC7A11 expression in the cisplatin group. However, SLC7A11 expression in the Cis + mPDA@DFO and Cis + FSH-mPDA@DFO groups increased to levels comparable to those of the control group (Figure 4B). These results suggest that both mPDA@DFO and FSH-mPDA@DFO effectively exert their pharmacological effects and inhibit ferroptosis in the ovary.

Given the iron-chelation properties of FSH-mPDA@DFO nanoparticles *in vivo*, we hypothesized that they could inhibit ferroptosis and protect against cisplatin-induced POF. As anticipated, ovarian volume and follicle count were significantly reduced in the cisplatin group. However, both were restored in the Cis + mPDA@DFO and Cis + FSH-mPDA@DFO groups (Figure 4C). We examined granulosa cell proliferation, which is essential for follicular development, was examined. Ki-67-positive granulosa cells were decreased in the cisplatin group but recovery to levels approaching those of the control group was observed in the Cis + mPDA@DFO and Cis + FSH-mPDA@DFO groups (Figure S9A). These results indicate that FSH-mPDA@DFO nanoparticles have a beneficial effect on ovarian morphology, follicular development, and cell proliferation.

The ovary is a key endocrine organ that regulates homeostasis by secreting steroid hormones. STAR, CYP17, and CYP19 are crucial for steroidogenesis. IF imaging showed disrupted estrous cycles and decreased expression of STAR, CYP17, and CYP19 following cisplatin treatment. Treatment with Cis + mPDA@DFO and Cis + FSH-mPDA@DFO restored ovarian endocrine function (Figure 4D, S9B-E). Cisplatin treatment significantly reduced AMH levels compared to the control group. Importantly, both mPDA@DFO and FSH-mPDA@DFO treatments markedly restored AMH concentrations, with FSH-mPDA@DFO achieving near-normalization to control levels (Figure S9F). We monitored mice over three estrous cycles using vaginal smears. The control group had a regular estrous cycle, while cisplatin-treated mice entered prolonged diestrus. Partial recovery of the estrous cycle was observed after treatment with mPDA@DFO or FSH-mPDA@DFO (Figure S9G). Ovarian aging is often associated with increased interstitial fibrosis 23. αSMA and collagen type III (Collagen 3) are markers of fibrosis 33. IF imaging revealed increased expression of αSMA and Collagen 3 in the cisplatin group, which was reduced to control levels following mPDA@DFO and FSH-mPDA@DFO treatment (Figure S10A-D). Granulosa cells play a crucial role in transmitting nutrients and signals to the oocytes, essential for follicular development and oocyte quality 34-36. Gap junction protein connexin 43 (CX43) and oocyte microvillar density marker radixin (RDX) are key to this communication. IF results showed that fluorescence intensities of CX43 and RDX decreased after cisplatin treatment. Treatment with mPDA@DFO and FSH-mPDA@DFO restored these levels to those seen in the control group (Figure 4E, S11A-B). These results suggest that FSH-mPDA@DFO nanoparticles effectively attenuate the impacts of cisplatin on ovarian endocrine homeostasis, fibrosis, and communication between granulosa cells and oocytes by lowering free iron concentrations in the cisplatin-induced POF mouse model.

To further investigate the therapeutic effects of FSH-mPDA@DFO at a microscopic level, RNA-seq was performed on ovarian tissues, followed by Mfuzz analysis. The results indicated significant increases in pathways related to ribosome biogenesis, ferroptosis, estrogen signaling, drug metabolism, and retinoic acid metabolism in the cisplatin group, which were restored after treatment. Concurrently, metabolic signaling pathways, lysosomal pathways, GnRH signaling pathways, and fatty acid metabolism were significantly downregulated in the cisplatin group. However, these pathways returned to control levels post-treatment, particularly in the FSH-mPDA@DFO group (Figure 4F). Collectively, these findings demonstrate that FSH-mPDA@DFO nanomaterials inhibit ferroptosis in granulosa cells and effectively treat cisplatin-induced POF.

### FSH-mPDA@DFO nanoparticles increase the oocytes quality the litter size of cisplatin induced POF mice

Oogenesis is another critical function of the ovary 37. Superovulation experiments showed that the cisplatin group had fewer ovulated oocytes and more abnormal oocytes. Treatment with mPDA@DFO and FSH-mPDA@DFO nanoparticles restored the number of ovulated oocytes to normal and reduced abnormal oocytes (Figure 4G, S12A-B). The organization and morphology of the meiotic spindle are crucial for accurate chromosome separation during meiosis I and meiosis II, and are key indicators of oocyte quality 38-39. Spindles were stained with α-Tubulin and DNA with DAPI to examine spindle morphology and chromosome alignment in aging oocytes. Most fresh metaphase I oocytes had typical barrel-shaped spindles with well-aligned chromosomes. However, structure and chromosome alignment were significantly disrupted in POF oocytes. As expected, treatment with mPDA@DFO and FSH-mPDA@DFO significantly reduced the incidence of abnormal spindle morphology. Accurate alignment of chromosomes is crucial for proper chromosome segregation in subsequent stages. We found that chromosomes were neatly aligned on the equatorial plate in fresh oocytes. However, oocytes in the cisplatin group displayed significant disorganization, with chromosomes not arranged in a linear array but rather distributed across a wide area (Figure 4G, S12C-D). Statistical analysis showed that the chromosome region width was significantly increased in cisplatin-treated oocytes (Control: 4.28 ± 0.94 μm; cisplatin: 11.14 ± 1.58 μm). This was effectively corrected by treatment with mPDA@DFO or FSH-mPDA@DFO (mPDA@DFO: 11.14 ± 1.58 μm; FSH-mPDA@DFO: 5.27 ± 0.79 μm) (Figure S12D). The quantity and quality of oocytes are crucial for successful pregnancy and species perpetuation 37. Furthermore, fertility tests revealed a significant reduction in the number of pups born to mice in the cisplatin group. However, following treatment with mPDA@DFO and FSH-mPDA@DFO, fertility rates rebounded, correlating with the results of the oocyte quality assessments (Figure 4G). Taken together, FSH-mPDA@DFO nanoparticles effectively improve both the quantity and quality of oocytes in cisplatin-treated mice and enhance the fertility in mice with cisplatin-induced POF.

To clarify the advantages of FSH-mPDA@DFO over mPDA@DFO, we have comprehensively evaluated therapeutic outcomes across multiple dimensions, including dosage efficiency, ovarian/follicle morphology, endocrine function, oocyte quantity/quality, and molecular profiling. Our quantitative analyses demonstrate that FSH-mPDA@DFO exhibits significantly enhanced protective effects compared to mPDA@DFO, including (1) Targeted delivery enabling 50% dose reduction; (2) Enhanced functional restoration of ovarian reserve (AMH), steroidogenesis (CYP17A1), and granulosa cell coordination (CX43); (3) Broad-spectrum efficacy in rectifying oocyte defects and ferroptosis-related molecular dysregulation.

### FSH-mPDA@DFO reduce TFRC expression to counteract ferroptosis

**Figure 5:**
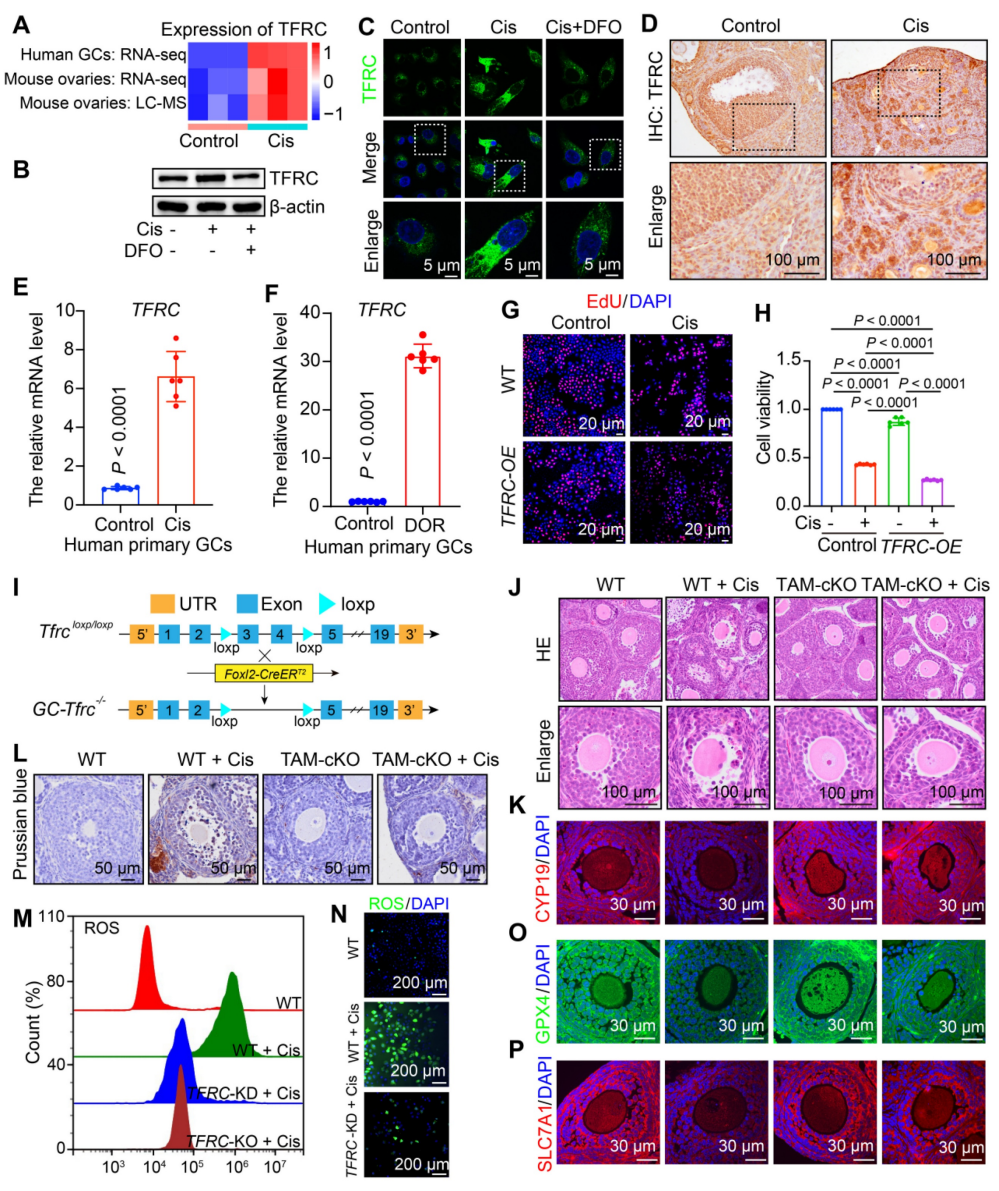
** Knockout of *Tfrc* in granulosa cells protects against cisplatin-induced premature ovarian failure. A** Heatmap showing the expression levels of TFRC derived from RNA-seq and LC-MS data. **B** Western blotting analysis of TFRC in granulosa cells treated with cisplatin and cisplatin + DFO. **C** IF staining of TFRC in granulosa cells treated with cisplatin and cisplatin + DFO. **D** IHC staining of TFRC in mouse ovaries treated with cisplatin. **E** Relative mRNA levels of *TFRC* in human primary granulosa cells treated with cisplatin. **F** Relative mRNA levels of *TFRC* in primary granulosa cells from patients with DOR. **G-H** EdU staining (G) and CCK8 assay for cell viability in control, cisplatin, *TFRC*-overexpressing (*TFRC*-OE), and cisplatin + *TFRC*-OE cells. **I** Schematic representation of the deletion of *Tfrc* exons 3 and 4 via *Foxl2-CreERT2*-mediated recombination in the granulosa cells of *GC-Tfrc^-/-^*. **J** HE staining of ovarian sections. **K** IF staining of CYP19 in ovarian sections. **L** Prussian blue staining of ovarian sections. **M-N** Flow cytometry and IF were employed to detect ROS content. **O-P** IF staining of GPX4 (O) and SLC7A11 (P) in ovarian sections.

Cisplatin induces iron overload in granulosa cells, leading to POF. We aim to explore the mechanism by which cisplatin chemotherapy disrupts iron homeostasis in ovarian granulosa cells. TFRC mediates iron import via extracellular transferrin and plays a key role in regulating cellular iron metabolism and maintaining iron homeostasis 12. TFRC mRNA and protein levels were significantly elevated in KGN cells, mouse ovaries, and human primary granulosa cells treated with cisplatin, consistent with RNA-seq and LC-MS results (Figure 5A-F). Due to insufficient granulosa cell collection in clinical patients with POF, we examined granulosa cells obtained from patients with diminished ovarian reserve (DOR). DOR is progressive and can advance to POF 1. Notably, the mRNA level of *TFRC* was elevated 30-fold in granulosa cells from DOR patients (Figure 5F). These findings suggest that TFRC is upregulated in both the POF mouse model and cisplatin-treated human granulosa cells, as well as in granulosa cells from DOR patients, indicating its significant role in regulating ferroptosis in POF.

To validate the role of TFRC in cisplatin-induced POF, we overexpressed *TFRC* in KGN cells, which inhibited cell viability. The viability of *TFRC*-overexpressing cells was further reduced following cisplatin treatment (Figure 5G-H, S13A). To investigate the functions of TFRC in follicular development and fertility maintenance, we constructed mice with a conditional knockout of exons 3 to 4 of the *Tfrc* gene specifically in granulosa cells (referred to as TAM-cKO mice) by crossing *Foxl2-CreERT2* transgenic mice with floxed mice and administering tamoxifen (TAM) as needed (Figure 5I). HE staining showed no significant changes in the morphology of TAM-cKO follicles compared to wild-type (WT) follicles. After cisplatin treatment, WT follicles displayed abnormal morphology and signs of atresia, whereas TAM-cKO follicles retained normal morphology and did not undergo atresia (Figure 5J). These findings suggest that the knockout of *Tfrc* in granulosa cells rescues cisplatin-induced follicular atresia. Subsequently, we examined granulosa cell steroidogenesis via IF staining of CYP19. After *Tfrc* knockout in cisplatin-treated granulosa cells, CYP19 expression increased compared to that in WT ovaries, indicating rescue of steroidogenesis in granulosa cells (Figure 5K, S14A), indicating that steroidogenesis in granulosa cells was rescued. Overall, these results suggest that cisplatin contributes to ovarian injury by upregulating TFRC. Excess Fe^2+^ promotes ferroptosis by increasing ROS levels, leading to oxidative stress and lipid peroxidation formation 40. Next, we detected changes in iron ion aggregation, ROS levels, lipid peroxidation, and glutathione. Prussian blue staining confirmed significant iron accumulation in granulosa cells of cisplatin-treated WT mice, but not in TAM-cKO mice (Figure 5M). ROS levels were elevated in WT cells after cisplatin treatment but decreased in *TFRC* knockout or knockdown cells (Figure 5N-O, S13B-F). This finding indicates that decreased TFRC expression inhibits excessive iron ion entry into the cells triggered by cisplatin, thereby reducing ROS production. We also assessed the protein levels of GPX4 and SLC7A11, finding no significant differences between the cKO and untreated WT groups. However, the fluorescence intensity of GPX4 and SLC7A11 was significantly reduced in cisplatin-treated WT mice, while it increased in cKO granulosa cells compared to cisplatin-treated WT mice (Figure 5O-P, S14B-C). This suggests that following TFRC knockout, lipid peroxidation processes and glutathione levels returned to normal, inhibiting ferroptosis.

To investigate whether DFO regulates TFRC expression, we examined TFRC protein levels in the cisplatin and cisplatin + DFO groups. TFRC exhibited strong fluorescence intensity in the cisplatin group and weak intensity in the cisplatin + DFO group. Western blotting confirmed that DFO reduced TFRC protein levels, consistent with IF findings (Figure 5B-C, S14D-E). This suggests that DFO can inhibit TFRC expression and reduce cellular iron ion entry.

### Lysosome-enriched FSH-mPDA@DFO nanoparticles inhibit ferritinophagy

**Figure 6:**
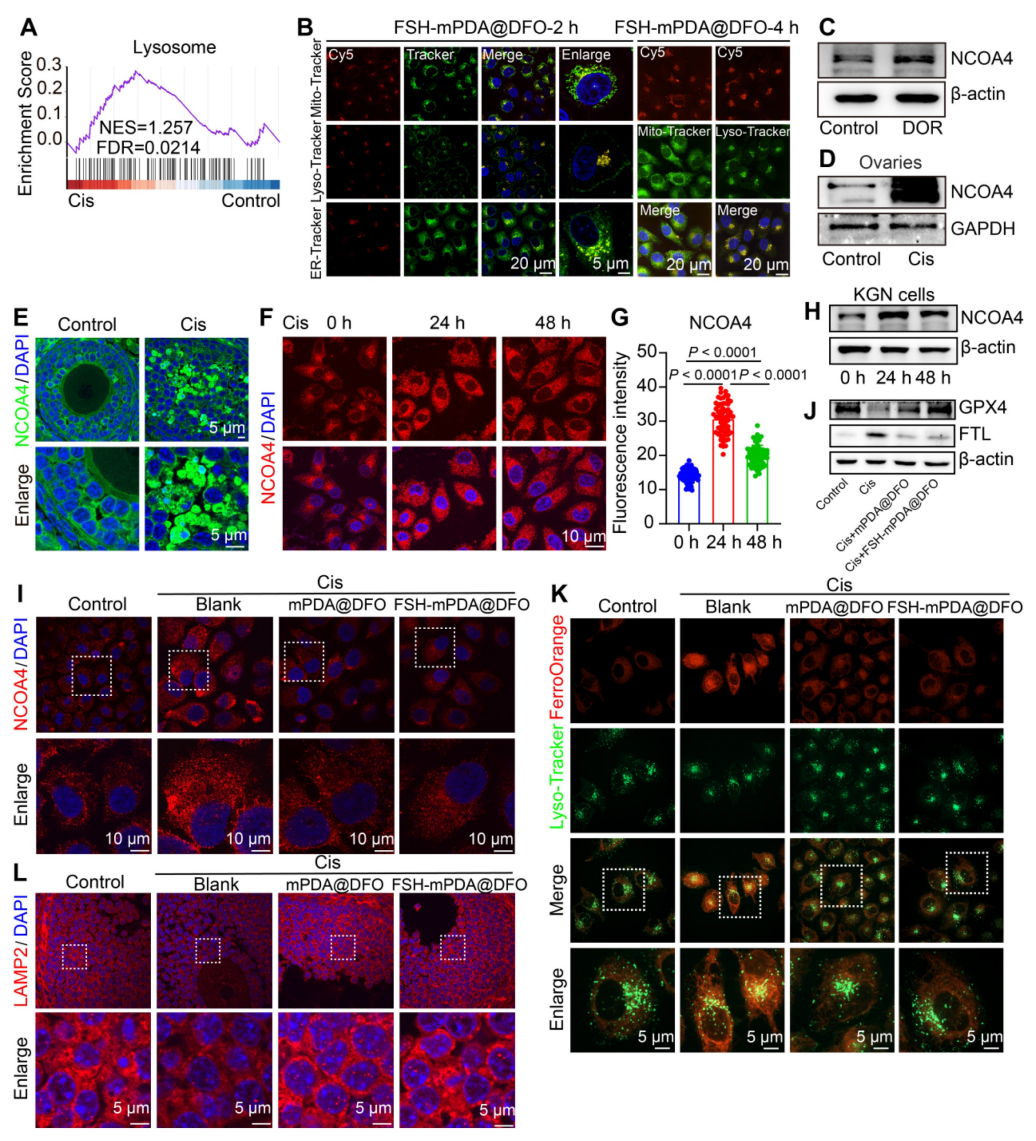
** FSH-mPDA@DFO nanoparticles inhibit ferritinophagy. A** GSEA enrichment analysis of RNA-seq data from human primary granulosa cells. **B** Colocalization of FSH-mPDA@DFO with different organelles, KGN cells were treated with FSH-mPDA@DFO for 2 or 4 h. **C** Western blotting was performed to detect the protein expression of NCOA4 in granulosa cells of DOR patients aged < 35 years. Infertile patients aged < 35 years due to male factors were used as controls. **D** Western blotting analysis of NCOA4 in cisplatin-treated mouse ovaries. **E-G** IF staining and fluorescence intensity analysis of NCOA4 in cisplatin-treated mouse ovaries (E) and KGN cells (F, G). **H** The western blotting analysis of NCOA4 in cisplatin-treated KGN cells. **I** The IF staining of NCOA4 within KGN cells. **J** The western blotting analysis of FTL and GPX4 in KGN cells treated with Cis or Cis + DFO. **K** Co-localization analysis of lysosomes (Lyso-Tracker Green) and intracellular iron (Ferro-Orange) in KGN cells treated with or without Cis, Cis + mPDA@DFO, or Cis + FSH-mPDA@DFO for 24 h. **L** IF staining of LAMP2 in ovarian tissue sections.

KEGG and GSEA analyses of RNA-seq data from cisplatin-treated human granulosa cells were enriched in the lysosome signaling pathway (Figure 1B, 6A). Confocal microscopy revealed substantial colocalization of FSH-mPDA@DFO nanoparticles with Lyso-Tracker, a lysosomal fluorescent dye (Figure 6B, S15A-E). This indicates that FSH-mPDA@DFO nanoparticles function within lysosomes, which are major organelles involved in cellular iron metabolism 41. Specifically, lysosome-mediated ferritin degradation, known as ferritinophagy, contributes to the intracellular labile iron pool and facilitates ferroptosis. Nuclear receptor coactivator 4 (NCOA4)-mediated ferritinophagy promotes ferroptosis by releasing free iron and increasing the labile iron pool (LIP) 42-43. NCOA4 was significantly upregulated in granulosa cells from patients with DOR, in cisplatin-treated KGN cells, and in mouse ovaries (Figure 6C-H), consistent with reports that NCOA4 promotes ferroptosis and leads to ferritin degradation and iron overload under cisplatin induction 43. We then examined the effect of FSH-mPDA@DFO's effect on NCOA4 expression. Treatment with FSH-mPDA@DFO significantly decreased NCOA4 levels to those of the control group (Figure 6I, S16A). Selective autophagy of ferritin, known as ferritinophagy, is required for iron release. Additionally, we evaluated the expression of the ferritin light chain (FTL). Cisplatin treatment significantly increased FTL protein levels, but they returned to near-normal after DFO treatment (Figure 6J, S16B-G), suggesting that FSH-mPDA@DFO regulates cellular iron metabolism. The iron released by ferritinophagy is thought to be primarily retained in lysosomes 42-43. Cisplatin increased lysosomal bioactive iron, as shown by increased FerroOrange colocalization with LysoTracker. FSH-mPDA@DFO reduced lysosomal bioactive iron levels significantly (Figure 6K). LAMP2 is critical for lysosomal membrane stability. Cisplatin significantly reduced LAMP2 levels, but FSH-mPDA@DFO restored them to normal (Figure 6L, S16H). Taken together, these results indicate that FSH-mPDA@DFO nanoparticles are enriched in lysosomes and inhibit ferritinophagy-mediated iron release.

### Mitochondria-enriched FSH-mPDA@DFO nanoparticles inhibit mitophagy in granulosa cells

**Figure 7:**
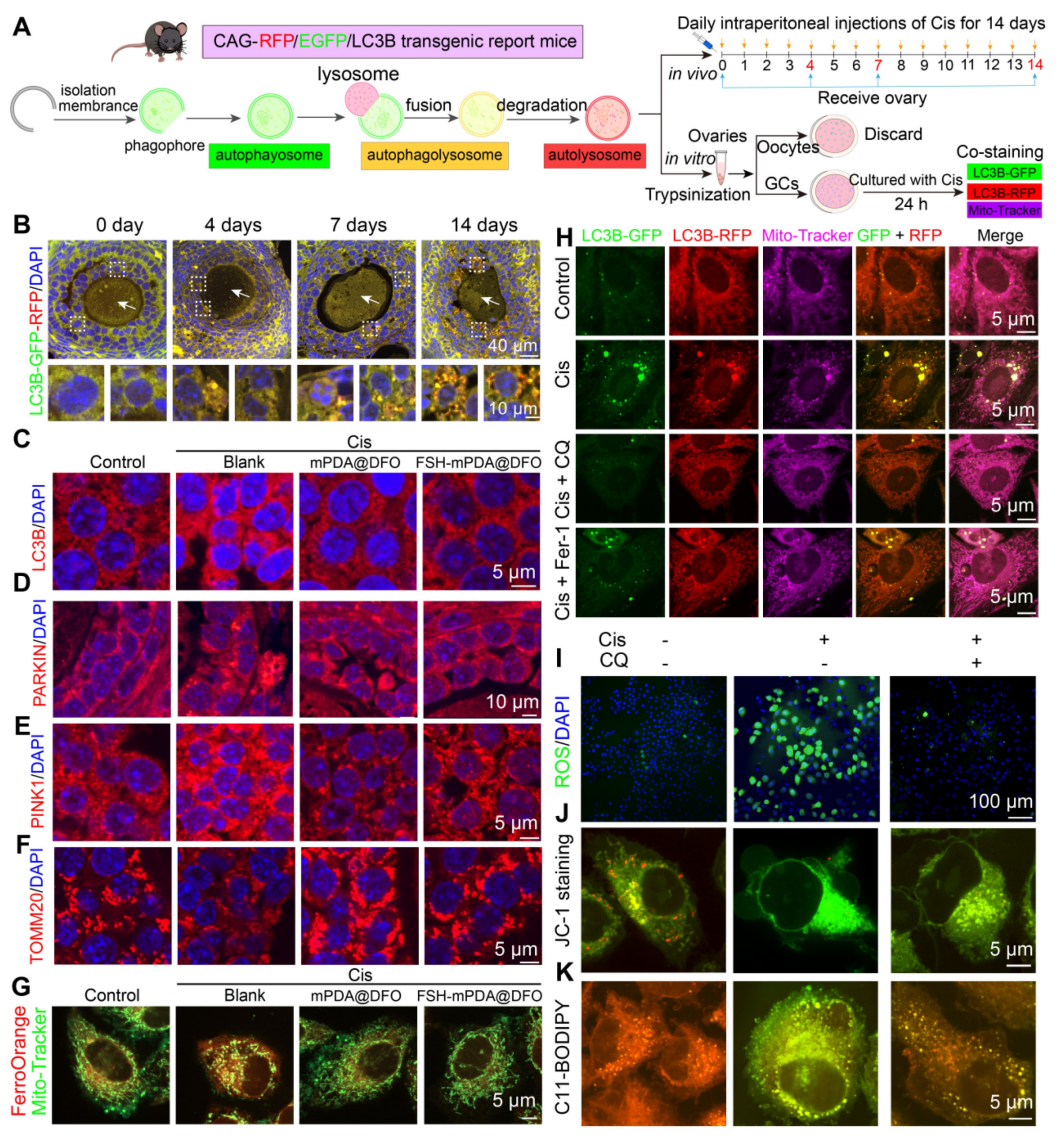
** Ferroptosis and mitophagy synergistically regulate cisplatin-induced POF. A** Schematic illustration of CAG-RFP-GFP-LC3B transgenic reporter mice used to detect changes in autophagic flux and observe patterns of mitophagy alterations both *in vivo* and *in vitro*. **B** Ovarian sections from CAG-RFP-GFP-LC3B transgenic reporter mice treated with cisplatin for 0, 4, 7 and 14 days. The white dashed box indicates the granulosa cells, and the white arrow POFnts to the oocyte. **C-F** IF staining of mitophagy-related markers (LC3B, PINK1, PARKIN, and TOMM20) expression in ovarian tissue sections. **G** The co-staining of Mito-Tracker-green and Ferro-Orange in KGN cells. These cells were treated with or without Cis, Cis + mPDA@DFO, or Cis + FSH-mPDA@DFO for 24 h. **H** Primary granulosa cells isolated from CAG-RFP-GFP-LC3B transgenic reporter mice were co-stained with Mito-Tracker. These cells were treated with or without Cis, Cis + CQ, or Cis + Fer-1 for 24 h. **I** Intracellular ROS levels were measured using a Reactive Oxygen Species Assay Kit. **J** The mitochondrial membrane potential was assessed with an enhanced mitochondrial membrane potential assay kit. **K** Lipid ROS levels were detected using C11-BODIPY 581/591 probes.

RNA-seq and LC-MS analyses showed elevated levels of PINK1-PARKIN-mediated mitophagy in the ovaries of cisplatin-treated mice (Figure S1K). To explore the role of mitophagy in cisplatin-induced ovarian injury, we examined mitophagy dynamics in ovarian granulosa cells using CAG-RFP/EGFP/LC3B transgenic reporter mice, a model for monitoring autophagy were utilized 19, 44. In this system, green puncta indicate autophagosomes, yellow puncta represent autophagolysosomes, and red puncta denote autolysosomes (Figure 7A). After cisplatin administration, granulosa cells showed a time-dependent increase in green, yellow, and red puncta over 4, 7, and 14 days, with red puncta predominating, indicating enhanced autophagic activity (Figure 7B, S17A). No autophagic signals were detected in oocytes, but a progressive rise in oocytes with abnormal morphology was observed (Figure 7B, S17B), correlating with increased follicular atresia (Figure S2K-L). To validate these findings, primary granulosa cells from CAG-RFP/EGFP/LC3B mice were treated with cisplatin in vitro. Co-staining with Mito-Tracker showed a significant increase in red puncta co-localized with mitochondria. Similarly, cisplatin-treated KGN cells had increased LC3B-positive puncta, some co-localized with Mito-Tracker (Figure S17C-F). Consistent with these observations, IF and western blotting analyses confirmed upregulation of mitophagy markers (PARKIN, PINK1, BNIP3, FUNDC1, and LC3B) and downregulation of p62 in cisplatin-treated mouse ovaries and KGN cells (Figure S18A-H). Furthermore, TEM provided ultrastructural evidence of increased mitophagy (Figure S18I-J). Collectively, these findings demonstrate that cisplatin chemotherapy induces mitophagy in granulosa cells, highlighting its potential role in cisplatin-associated ovarian damage.

The crosstalk between autophagy and ferroptosis amplifies their respective signaling pathways to regulate physiological and pathological processes 43-46. Confocal microscopy imaging revealed colocalization of FSH-mPDA@DFO with mitochondria (Figure 6B, S15C-D). To investigate the effect of DFO and FSH-mPDA@DFO on mitophagy, IF and western blotting were performed. DFO or FSH-mPDA@DFO inhibited the mitophagy pathway in cisplatin-treated KGN cells and mice, indicated by reduced levels of LC3B, PINK1, PARKIN, and TOMM20 compared to the cisplatin group (Figure 7C-F, S19A-C). Similarly, the addition of Fer-1 to cisplatin-treated cells also suppressed mitophagy, evidenced by decreased levels of PARKIN and BNIP3 (Figure S19D-G). Cisplatin treatment increased mitochondrial bioactive iron, shown by enhanced FerroOrange colocalization with Mito-Tracker. This effect was significantly reduced by FSH-mPDA@DFO supplementation (Figure 7G). Together, these results indicate that FSH-mPDA@DFO accumulates in mitochondria and inhibits mitophagy-mediated iron release.

We next examined the changes in ferroptosis following autophagy inhibition. Primary granulosa cells isolated from CAG-RFP/EGFP/LC3B mice were treated with Cis, Cis + CQ, or Cis + Fer-1, followed by co-staining with Mito-Tracker to assess mitophagy (Figure 7A). Autophagy inhibition in the Cis + CQ group reduced mitophagy levels to control levels, with effects similar to the Cis + Fer-1 group (Figure 7H). Importantly, autophagy inhibition in the Cis + CQ group also decreased ferroptosis levels, indicated by reduced ROS content, restored mitochondrial membrane potential, decreased lipid peroxidation, increased glutathione, and reduced iron ions (Figure 7I-K, S19H-M). These findings suggest that autophagy and ferroptosis synergistically regulate cisplatin-induced premature ovarian failure. Our further findings demonstrate that while targeting autophagy provides some protective effects, it is insufficient for full recovery. *In vitro*, co-treatment of KGN granulosa cells with cisplatin and CQ resulted in only a partial restoration of cell viability compared to cisplatin alone (Figure S20A). *In vivo*, mice receiving cisplatin + CQ showed a significant increase in ovarian volume compared to either cisplatin-only or CQ-only groups. However, this volume remained smaller than the control group (Figure S20B-D). HE staining confirmed partial restoration of follicle morphology, reducing cisplatin-induced atresia (Figure S20E). Superovulation assays showed reduced oocyte yield and increased abnormal oocytes in the cisplatin group. CQ alone also reduced ovulation. Co-treatment significantly increased oocyte yield compared to cisplatin alone but remained below control levels, and partially restored spindle assembly (Figure S20F-G). Furthermore, analysis of key markers (CX43 for gap junctions; CYP17, CYP19, STAR for steroidogenesis) showed that cisplatin + CQ significantly increased their expression compared to cisplatin or CQ alone, yet expression levels remained significantly lower than the control group (Figure S20H-O). Notably, this partial restoration contrasts sharply with the effects of the ferroptosis inhibitor deferoxamine (DFO). *In vitro*, DFO completely restored KGN cell viability. *In vivo*, DFO treatment effectively restored ovarian volume, normalized follicle counts and morphology via HE staining, fully rescued ovulation number and oocyte quality in superovulation assays, and brought the expression levels of CX43, CYP17, CYP19, and STAR back to control levels. This compelling efficacy led us to focus our primary investigation on DFO's therapeutic potential against cisplatin-induced ovarian damage and premature ovarian failure.

**Figure 8:**
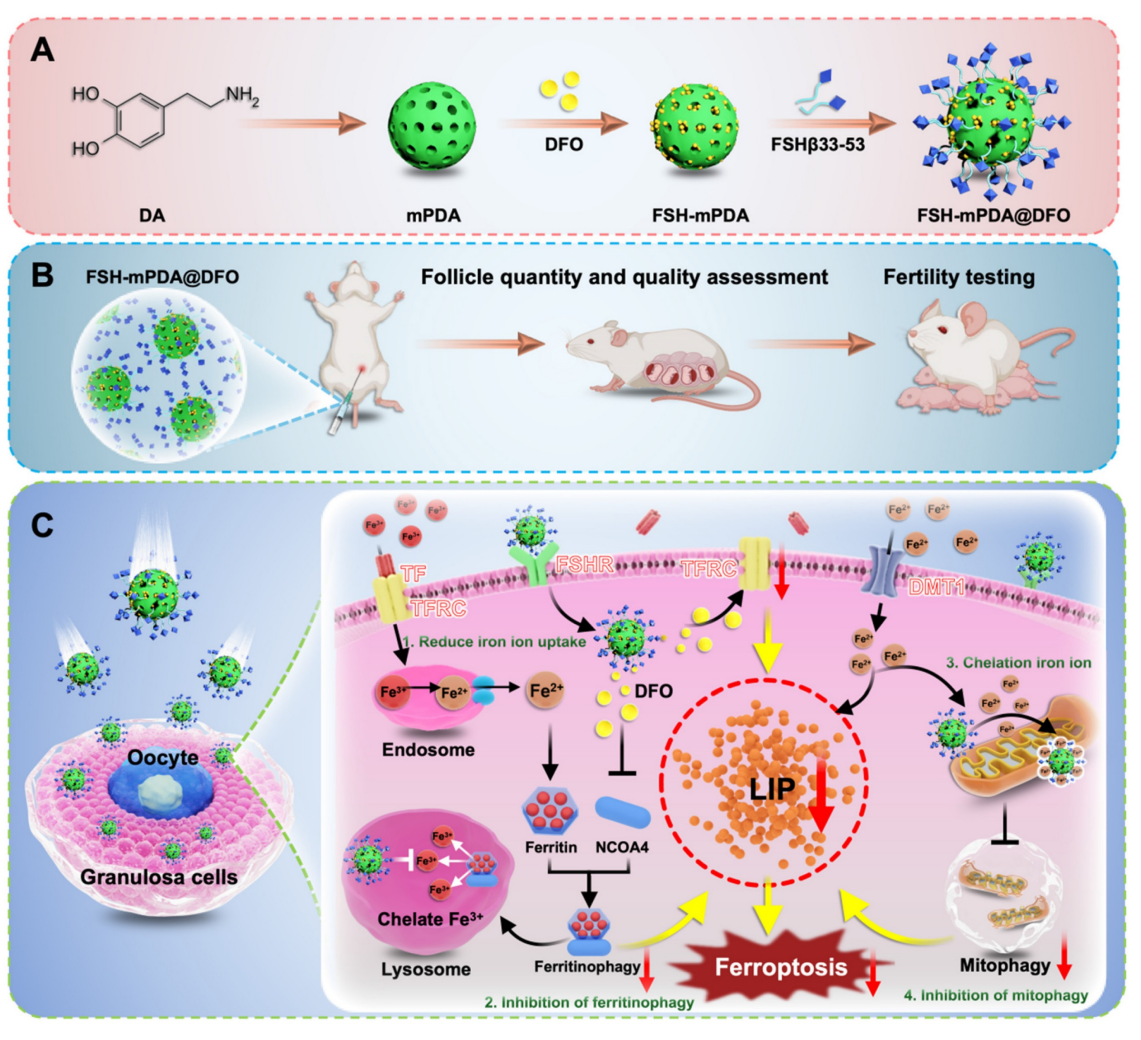
** The schematic diagram of FSH-mPDA@DFO therapeutic modality for premature ovarian failure. A** Visualization of the fabrication process of FSH-mPDA@DFO. **B** Schematic diagram of the therapeutic effect of FSH-mPDA@DFO on cisplatin-induced premature ovarian failure. **C** Schematic of the proposed mechanism FSH-mPDA@DF in granulosa cells, (1) inhibition of TFRC-mediated iron uptake, (2) inhibition of NCOA4-mediated ferritinophagy, (3) chelation of mitochondrial iron ions, (4) inhibition of mitophagy, thereby ameliorating LIP overload and regulating iron metabolism to prevent ferroptosis.

In summary, FSH-mPDA@DFO nanoparticles alleviate chemotherapy-induced ovarian damage by reducing iron uptake, chelating iron ions, inhibiting mitophagy, and blocking ferroptosis (Figure 8).

## Conclusion and Discussion

Chemotherapeutic agents, while significantly improving patient survival and prognosis, exert substantial toxic effects on normal tissues, particularly threatening fertility. In female patients, these agents induce granulosa cell damage within ovarian follicles, disrupt endocrine function, and impair oocyte quantity and quality, ultimately leading to POF and early menopause. Our transcriptomic and proteomic analyses revealed that cisplatin-induced POF is a ferroptosis-related disorder, commonly observed in chemotherapy-treated cancer patients. Despite its clinical prevalence, no effective therapeutic strategy exists to mitigate cisplatin-induced POF, limiting the overall benefits of chemotherapy 1-3. Although both chemotherapy-induced POF and cancer are associated with ferroptosis, their treatment requires opposing regulatory approaches. While small-molecule antioxidants and the ferroptosis inhibitor DFO have shown promise in alleviating symptoms, their use risks interfering with chemotherapy, which often relies on ferroptosis induction for efficacy.

Excessive iron accumulation, a key driver of ferroptosis, induces oxidative stress and cellular damage. The labile iron pool, a dynamic compartment of redox-active iron, is tightly regulated through iron uptake, utilization, distribution, and export. Transferrin receptor 1 (TFR1) mediates cellular iron uptake, while lysosomes supply iron to the labile iron pool via ferritin degradation and Fe^3+^ reduction 47. Mitochondria, which acquire iron from the cytosolic labile iron pool and lysosomes, serve as major sites of iron utilization and accumulation, contributing to the mitochondrial labile iron pool 41, 47. Mitochondrial fragmentation and enhanced mitophagy further release iron ions, sensitizing cells to ferroptosis. Elevated labile iron pool levels promote Fenton reactions, exacerbating chemical reactivity and cytotoxicity. Thus, strategies targeting iron accumulation and iron-dependent ferroptosis may offer therapeutic potential for chemotherapy-induced POF.

In this study, we elucidated the role of iron-dependent ferroptosis in follicular atresia, endocrine dysfunction, and fertility decline in cisplatin-induced POF through transcriptomic and proteomic profiling of human granulosa cells and mouse ovaries. We developed a multifunctional nanoregulator, FSH-mPDA@DFO, by encapsulating DFO within mesoporous polydopamine nanoparticles functionalized with an ovarian granulosa cell-targeting peptide (FSHβ33-53). Leveraging its iron-chelating and antioxidant properties, FSH-mPDA@DFO effectively reversed ferroptosis in POF cell models, mouse models, and human granulosa cells, demonstrating excellent biocompatibility. Mechanistically, FSH-mPDA@DFO is internalized primarily via macropinocytosis and dynamically responds to intracellular labile iron pool levels. It downregulates TFRC expression to reduce iron uptake, accumulates in lysosomes and mitochondria, inhibits ferritinophagy-mediated iron mobilization, and suppresses mitophagy, collectively ameliorating labile iron pool overload and restoring iron homeostasis. Additionally, FSH-mPDA@DFO mitigates ROS accumulation and counteracts lipid peroxidation.

Functionally, FSH-mPDA@DFO significantly attenuated ovarian damage, improved oocyte quantity and quality, and restored fertility and endocrine homeostasis in mice by modulating the labile iron pool and inhibiting ferroptosis. These findings highlight its potential to broaden the therapeutic window of chemotherapy, enabling patients to achieve optimal treatment outcomes. As a multifaceted ferroptosis inhibitor, FSH-mPDA@DFO represents a promising therapeutic strategy for chemotherapy-induced POF. Given its facile synthesis, targeted delivery, and biocompatibility, FSH-mPDA@DFO holds great potential for preventing and treating cisplatin-induced POF and other ferroptosis-related disorders in chemotherapy patients, ultimately enhancing therapeutic efficacy.

## Supplementary Material

Supplementary figures and tables.
