# Supplementary material for: Targeting granulosa cells with engineered DFO nanoparticles for the treatment of chemotherapy-induced premature ovarian failure
Source: Theranostics. 2025 Jul 4;15(15):7820–40. doi: 10.7150/thno.115416 (PMC12316035; doi:10.7150/thno.115416)
Supplement: Supplementary file 1 — Supplementary figures and tables. [file thnov15p7820s1.pdf]

Supplementary Materials

Supporting figure legend

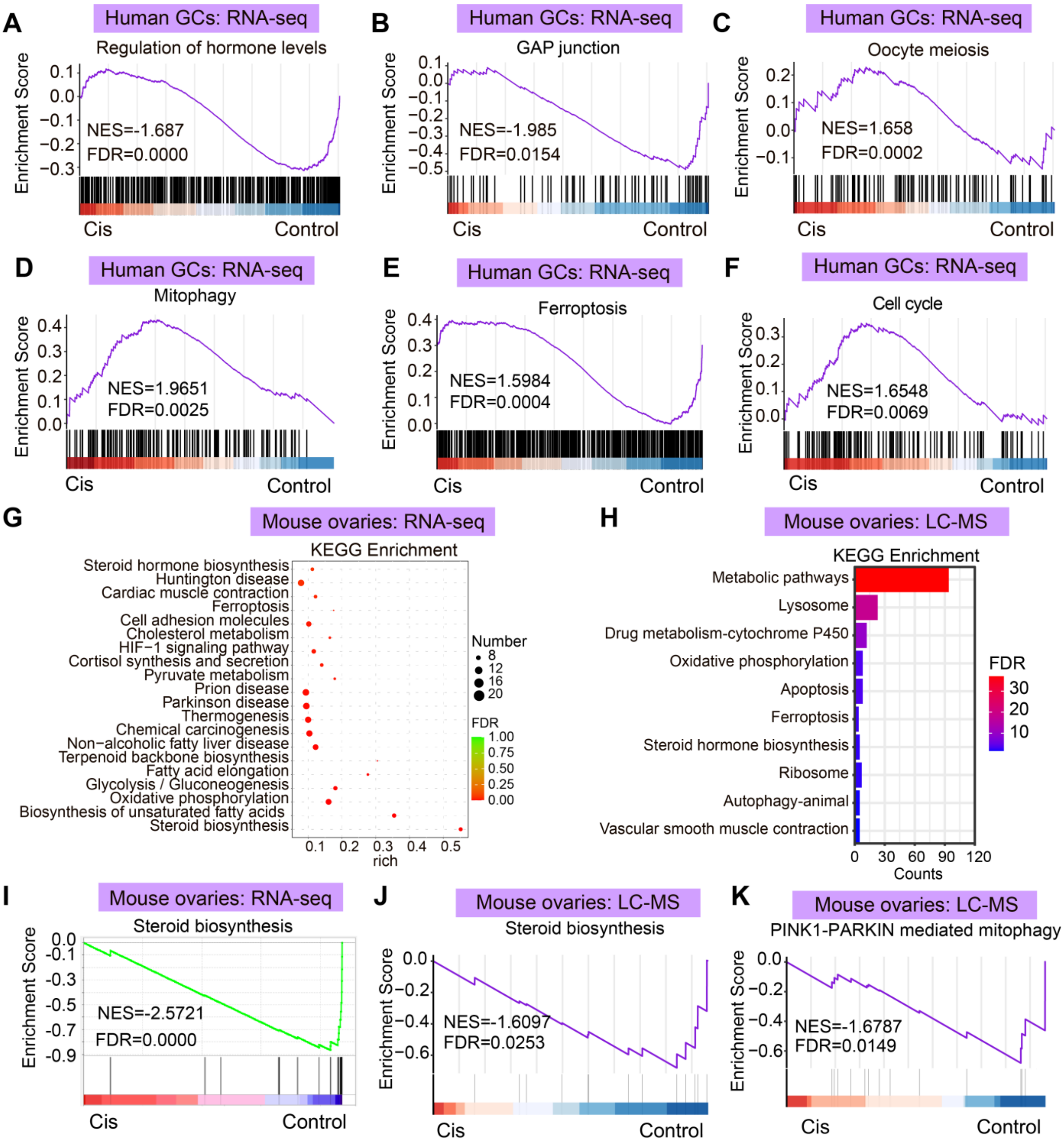

Figure S1 Cisplatin induces decreased steroid biosynthesis, increased ferroptosis, and elevated mitophagy

A-F GSEA enrichment analyses of RNA-seq data from cisplatin-treated human primary granulosa cells. **G-K** KEGG (G-H) and GSEA (I-K) enrichment analyses of RNA-seq data and LC-MS data from cisplatin-treated mouse ovaries.

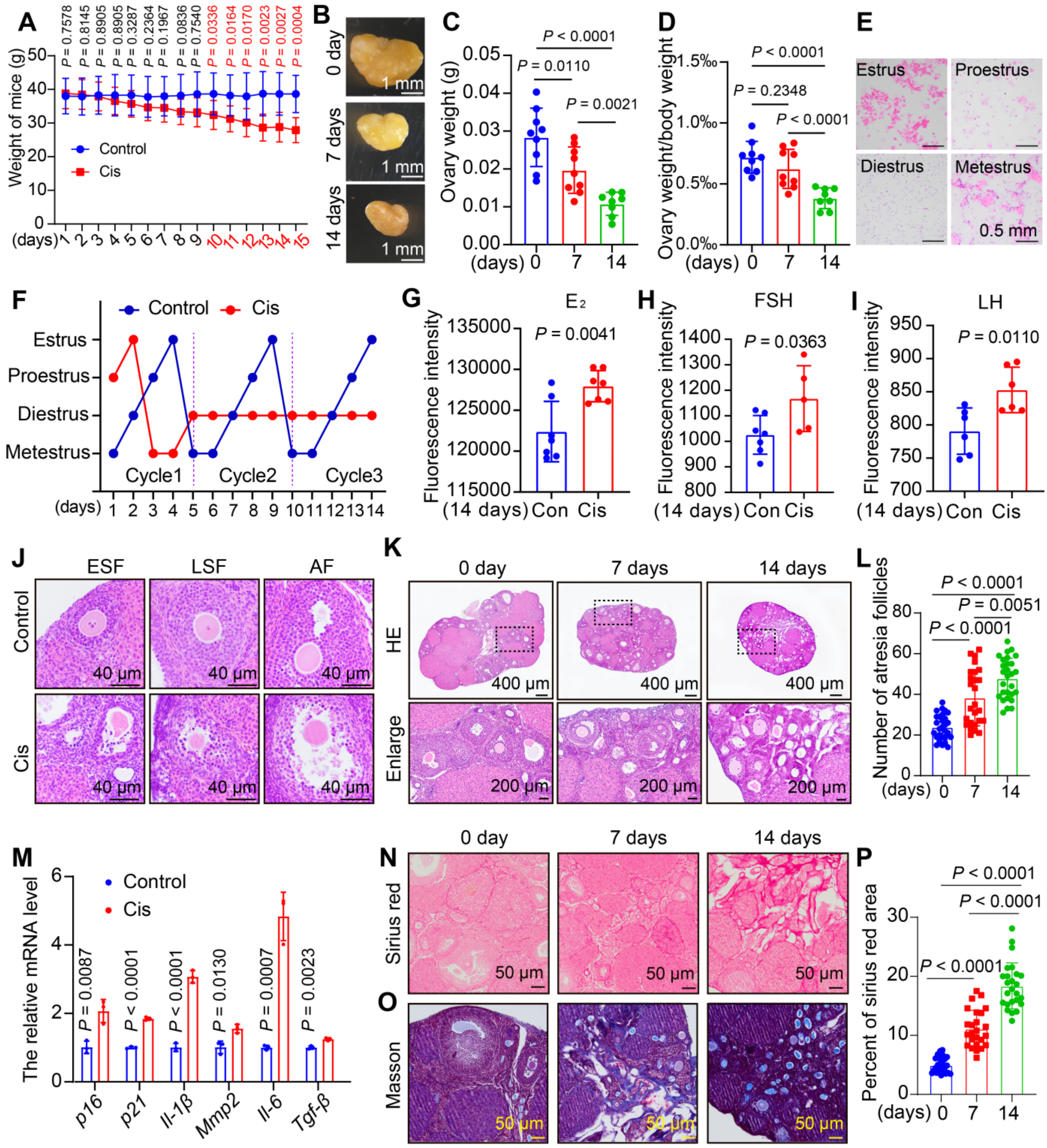

Figure S2 Cisplatin induces POF in mice

A Body weight of mice treated with cisplatin from day 1 to day 15. **B-D** Ovarian morphology (B), ovary weight (C), and the ratio of ovary weight to body weight (D) in mice treated with cisplatin at day 0, day 7, and day 14. **E** Vaginal smears taken during different phases of the estrous cycle. **F** Estrous cycle variations in mice were monitored for 14 consecutive days, covering approximately three full estrous cycles. **G-I** Serum concentrations of E_2, FSH, and LH in mice treated with cisplatin for 14 days. **J** HE staining of ovarian sections, including ESF,

LSF, and AF, from mice treated with cisplatin for 14 days. **K** HE staining of ovarian sections from mice treated with cisplatin for 0, 7, and 14 days. **L** Number of atresia follicles. **M** The relative mRNA levels of SASP-related genes. **N-P** Sirius red staining (N) and Masson's trichrome staining (O) of ovarian sections from mice treated with cisplatin. The percentage of Sirius red-stained area in ovarian sections from cisplatin-treated mice (P).

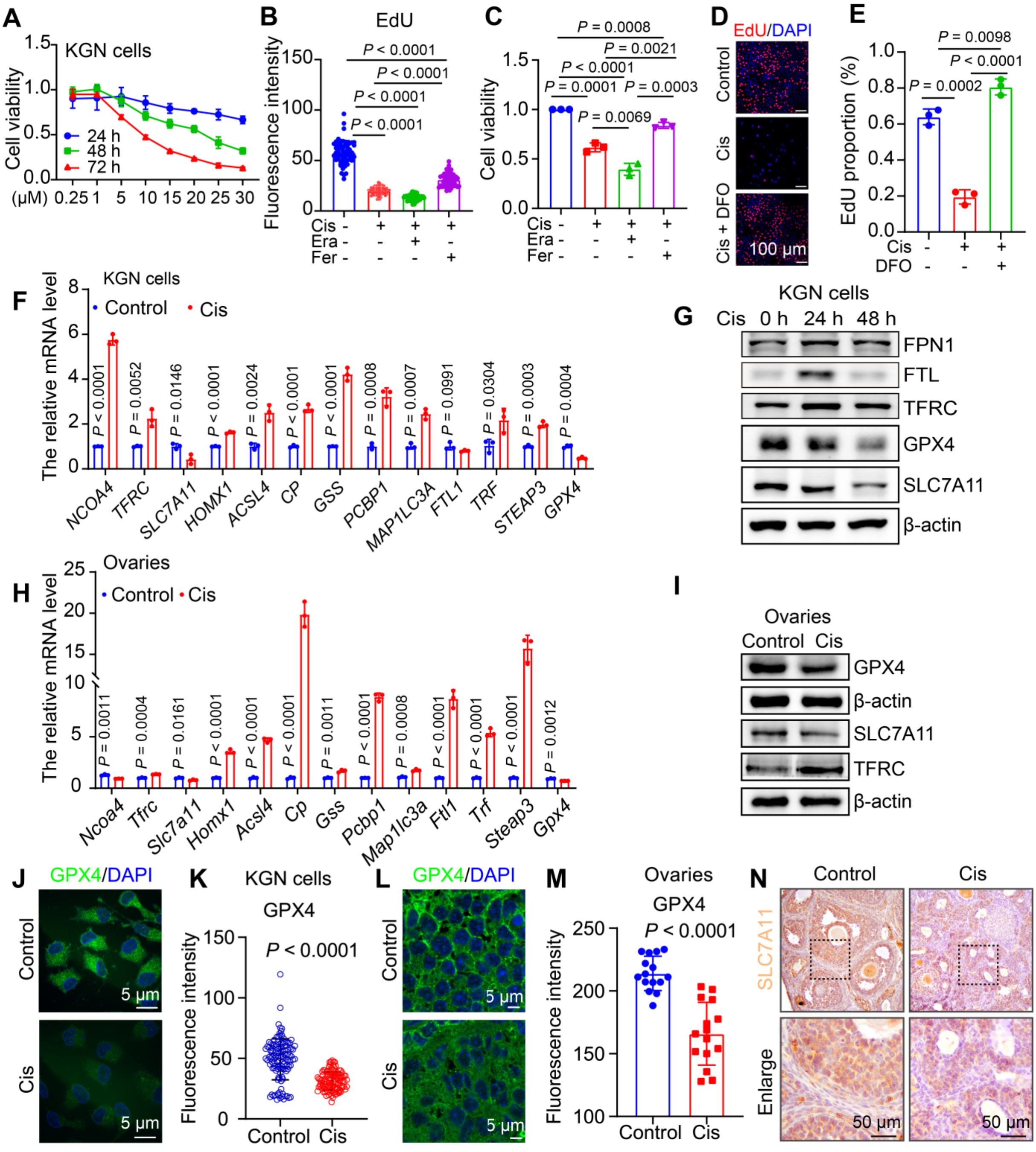

Figure S3 Cisplatin induces ferroptosis in granulosa cells

A Cell viability assessed via CCK8 assay. **B** The statistical analysis of EdU staining. **C** Cell viability assessed via CCK8 assay. **D-E** EdU staining and statistical analysis of KGN cells. **G** Lipid peroxidation measured using BODIPY 488/561 C11 staining. **F-I** The relative mRNA and protein levels regulating ferroptosis in cisplatin-treated KGN cells (F, G) and mouse ovaries (H, I). **J-M** IF staining and fluorescence intensity analysis of GPX4 in cisplatin-treated KGN cells (J, K) and mouse ovaries (L, M). **N** IHC staining of SLC7A11 in cisplatin-treated mouse ovaries.

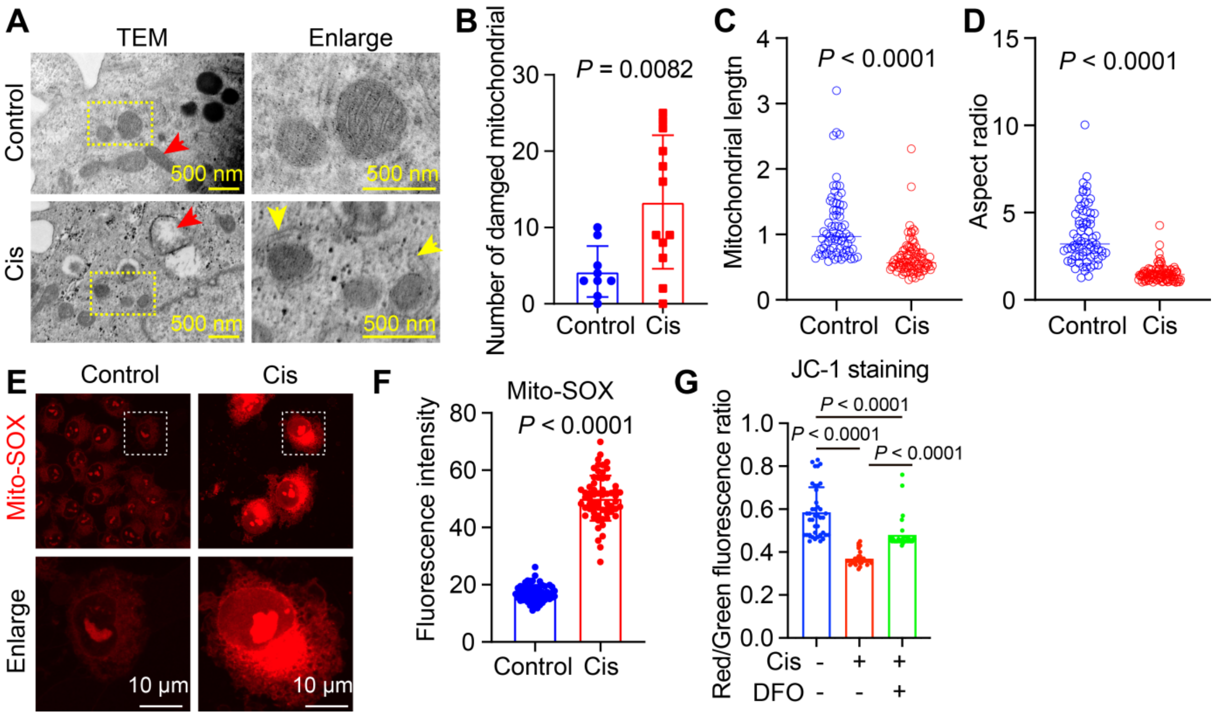

Figure S4 Cisplatin causes mitochondria damage in granulosa cells

A-D Transmission electron microscopy to observe ultrastructure changes in mitochondria (A). The statistical data about number of damaged mitochondrial (B) mitochondrial length (C) and mitochondrial aspect ratio (D). **E-F** The mitochondrial superoxide were detected with Mitochondrial Superoxide Assay Kit with Mito-SOX Red. **G** The ratio of red/green fluorescence about JC-1 staining.

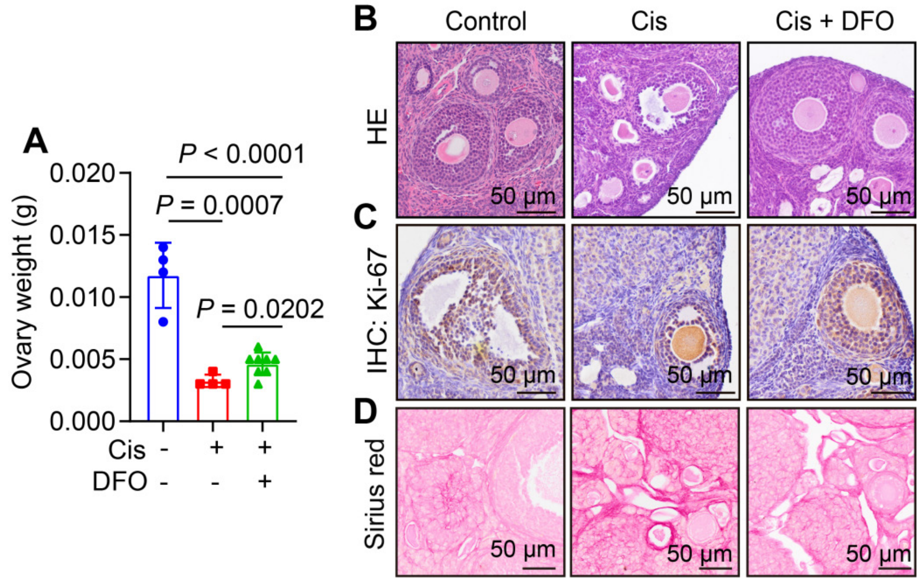

Figure S5 Cisplatin causes ovarian damage via ferroptosis

A Ovarian weights. **B-D** HE, IHC and Sirius red staining analysis of ovarian sections from mice treated with cisplatin or cisplatin + DFO for 14 days, respectively.

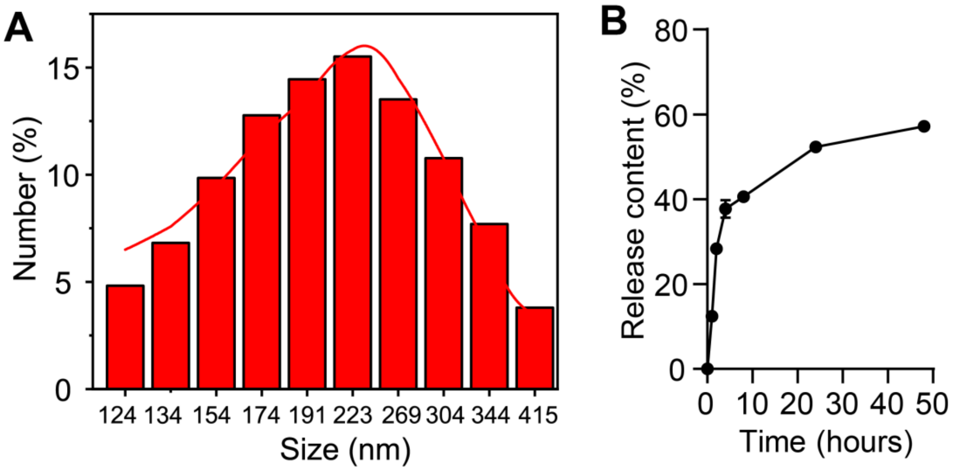

Figure S6 Physicochemical properties of FSH-mPDA@DFO nanoparticles

A Diagram of particle size analysis of FSH-mPDA@DFO. **B** The release content of FSH-mPDA@DFO.

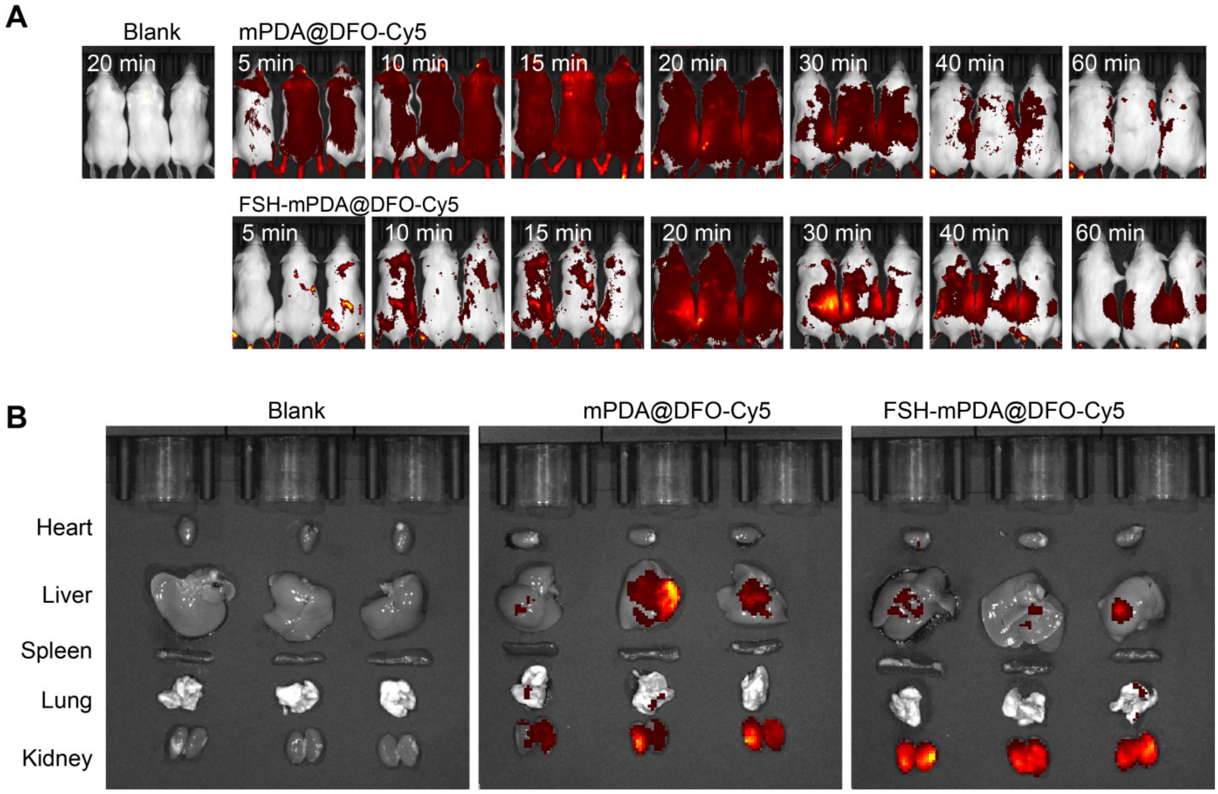

Fig. S7 FSH-mPDA@DFO nanoparticles specifically targeted ovarian granulosa cells for drug delivery

A-B *In vivo* biodistribution analysis of CD-1 mice showing fluorescence imaging of major organs and ovarian tissues at different time point post intravenous administration of saline, mPDA@DFO-Cy5 or FSH-mPDA@DFO-Cy5. Major organs and ovarian tissues are collected and pictured at 60 min after injection.

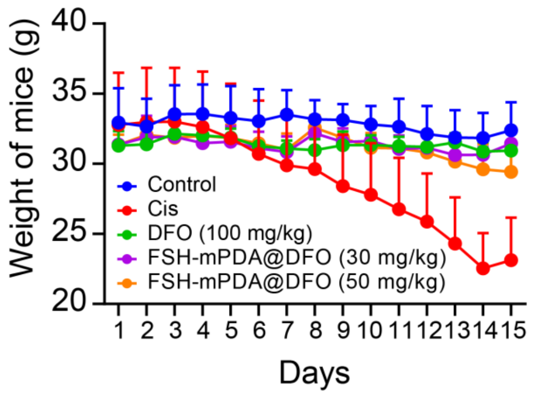

Figure S8 Body weight monitoring during 14-day treatment period with DFO or FSH-mPDA@DFO.

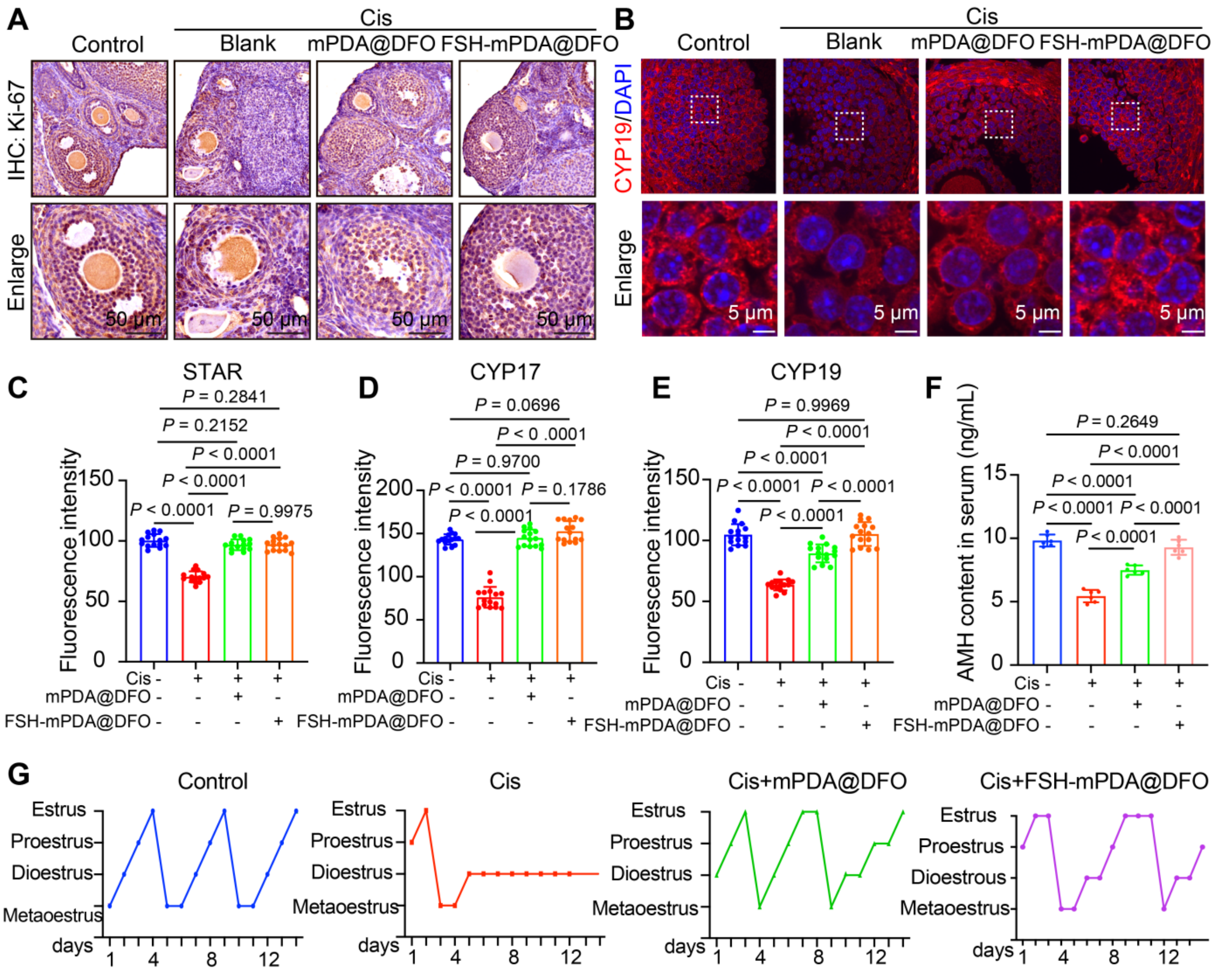

Figure S9 FSH-mPDA@DFO nanoparticles promoted granulosa cell proliferation and protected ovarian endocrine homeostasis in Cis-POF mice

A IHC staining of Ki-67 within ovarian sections. The mice were treated with Cis, Cis + mPDA@DFO, Cis + FSH-mPDA@DFO. **B-E** IF staining and statistical analysis of steroidogenic markers (STAR, CYP17, and CYP19) expression in ovarian tissue sections. **F** Quantification of AMH levels in mouse serum by ELISA. Mice were intraperitoneally administered Cis, Cis + mPDA@DFO, or Cis + FSH-mPDA@DFO for 14 days, and serum AMH levels were measured by ELISA. **G** Longitudinal monitoring of estrous cycle patterns over 14 consecutive days in mice treated with Cis, Cis + mPDA@DFO, and Cis + FSH-mPDA@DFO.

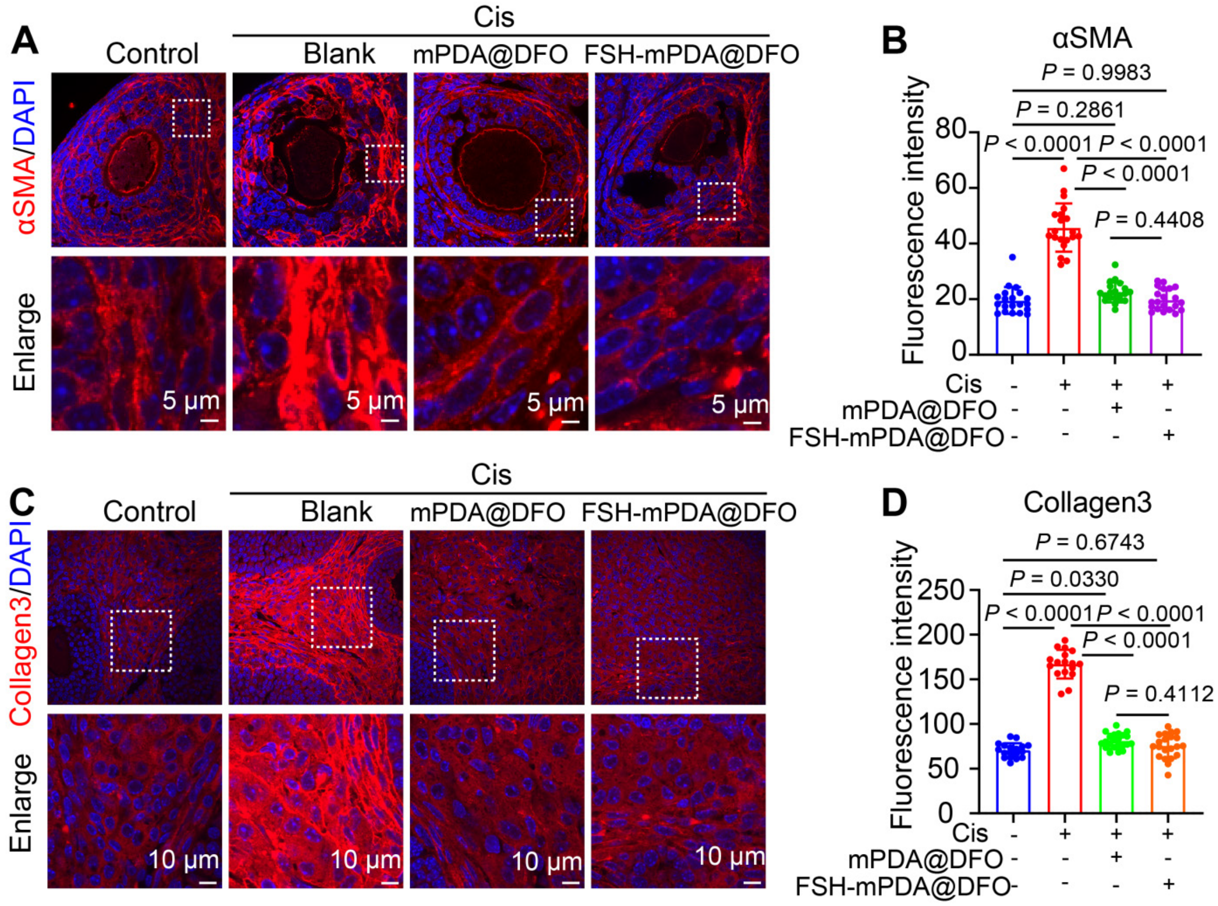

Figure S10 FSH-mPDA@DFO nanoparticles attenuate interstitial fibrosis in Cis-POF mice

A-D IF analysis and statistical analysis of interstitial fibrosis markers (αSMA and Collagen3) expression in ovarian tissue sections. The mice were treated with Cis, Cis + mPDA@DFO, Cis + FSH-mPDA@DFO.

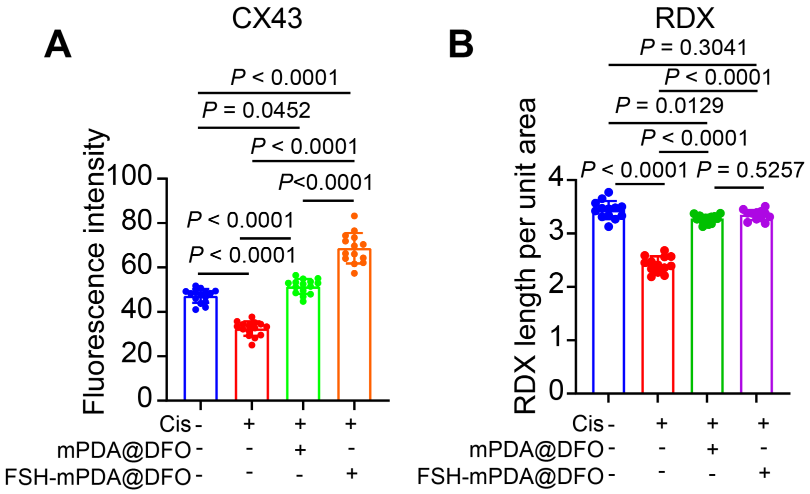

Figure S11 FSH-mPDA@DFO nanoparticles protect communicate between granulosa cells and oocytes

A-B The statistical analysis of gap junction marker (CX43) and microvillus marker (RDX) expression in ovarian tissue sections. The mice were treated with Cis, Cis + mPDA@DFO, Cis + FSH-mPDA@DFO.

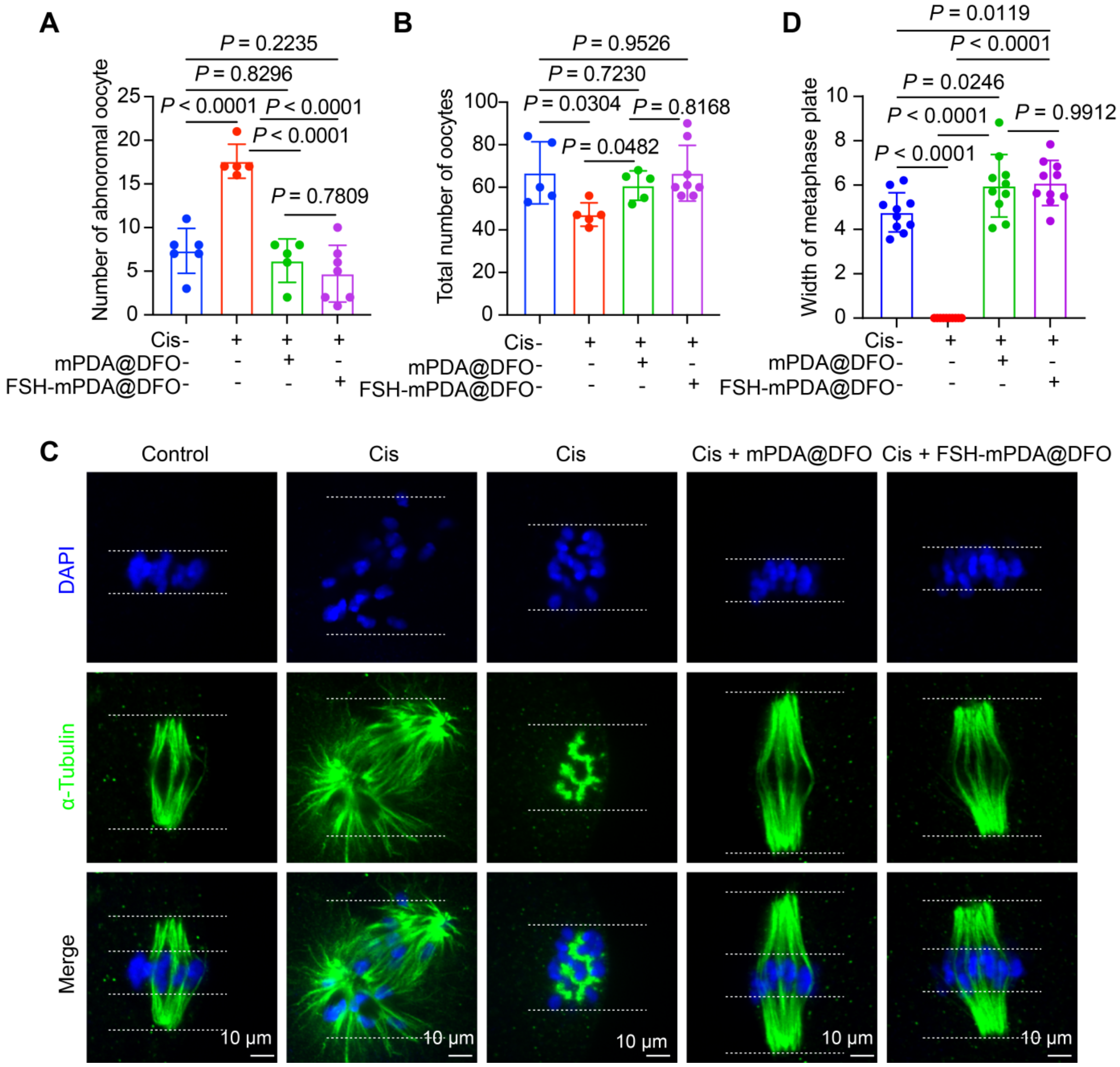

Figure S12 FSH-mPDA@DFO nanoparticles protect oocyte quality and quantity and quality

A-B The number abnormal oocytes (A) and total oocytes (B). **C** IF staining of α-Tubulin and DAPI within oocytes. **D** The width of metaphase plate within oocytes. The mice were treated with Cis, Cis + mPDA@DFO and Cis + FSH-mPDA@DFO.

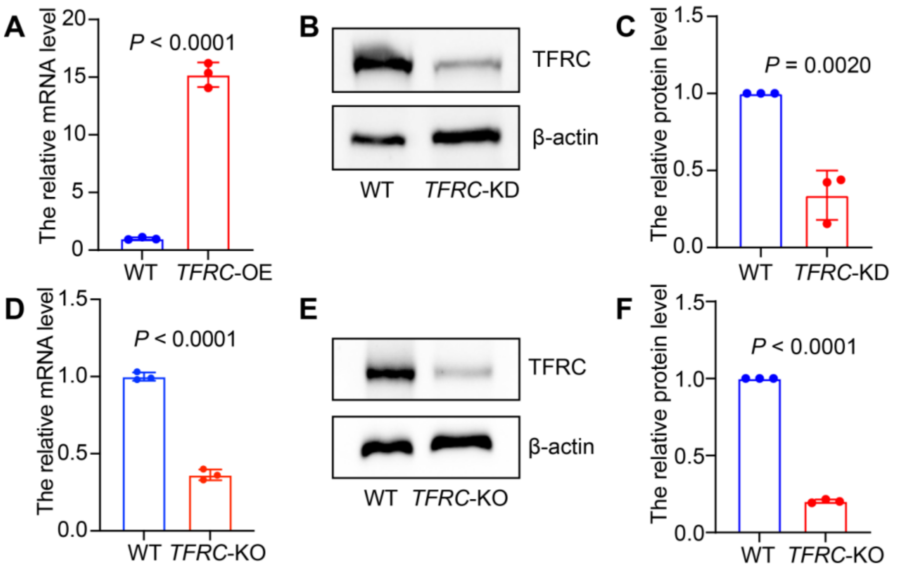

Figure S13 Validation of gene intervention efficiency

A The relative mRNA level of *TFRC* in *TFRC*-OE KGN cells. **B-C** The relative protein level of TFRC in *TFRC*-KD KGN cells. **D-F** The relative mRNA (D) and protein (E-F) level of TFRC in *TFRC*-KO KGN cells.

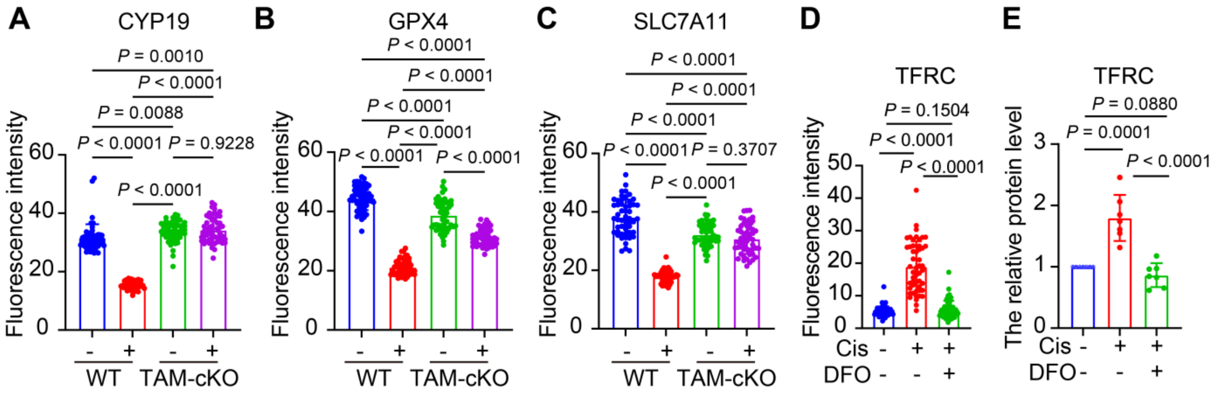

Figure S14 Conditional knockout of *Tfrc* in granulosa cells protects against cisplatin-induced ovarian damage

A-C Statistical analysis of the expression levels of CYP19, GPX4 and SLC7A11 in ovarian tissue sections. **D-E** Statistical analysis of the expression levels of TFRC.

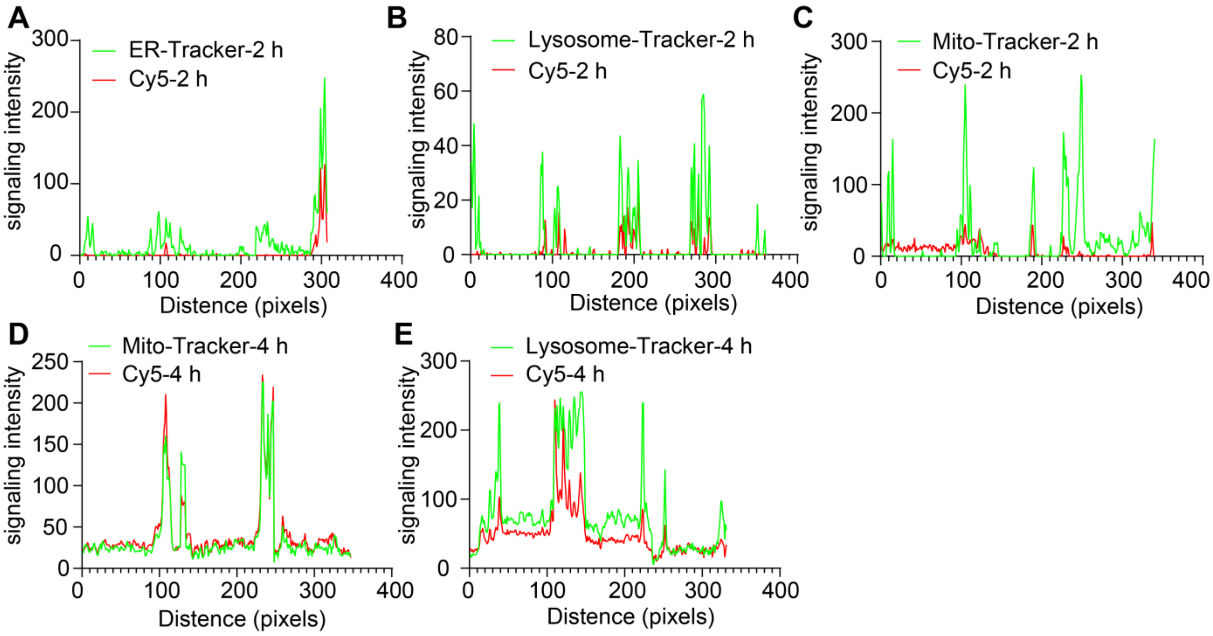

Figure S15 Co-localization analysis of FSH-mPDA@DFO-Cy5 and organelles

A-E The relative signal intensity of co-localizations by line scan after incubate with FSH-mPDA@DFO-Cy5 for 2 hours (A-C) and 4 hours (D-E).

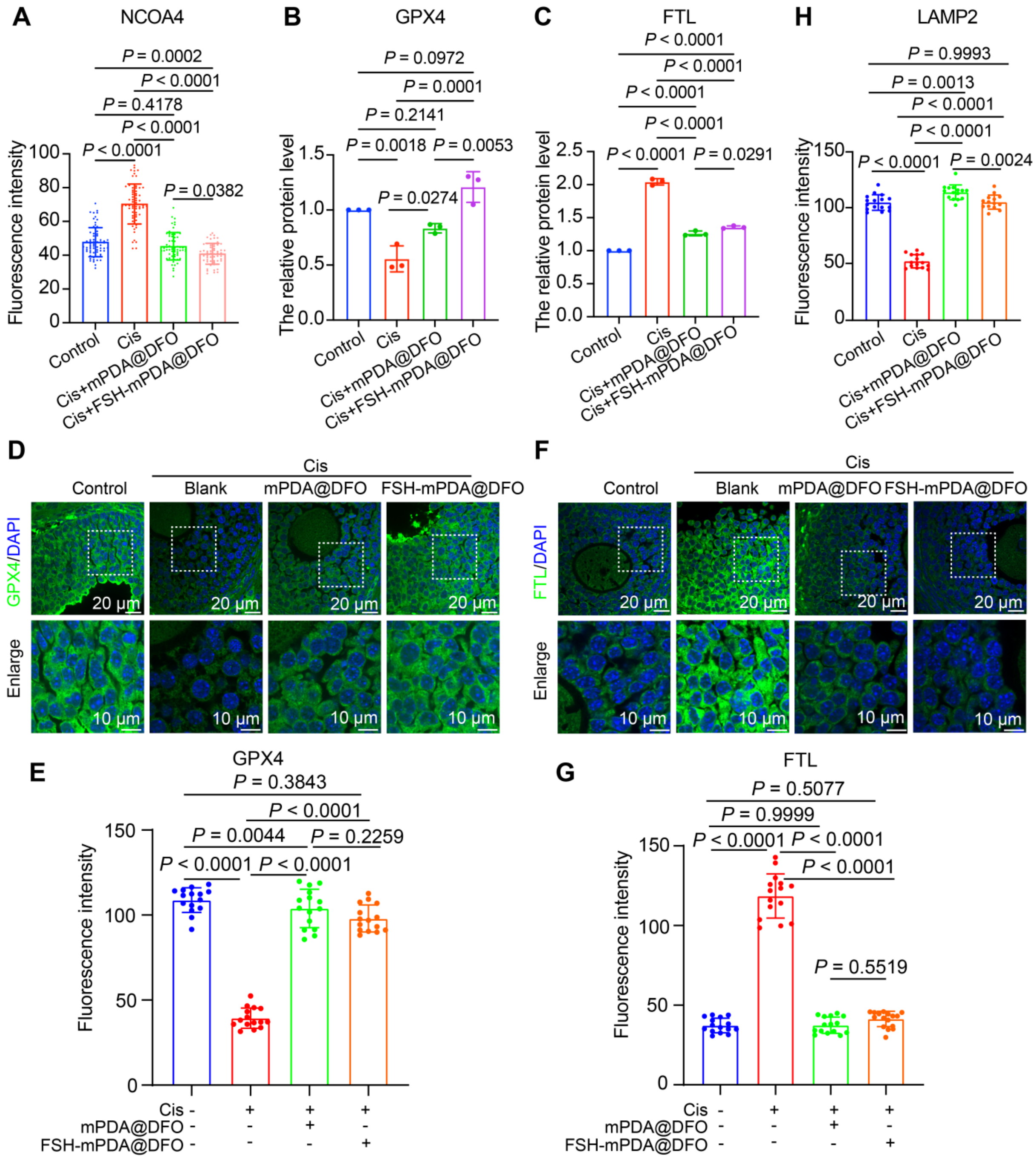

Figure S16 FSH-mPDA@DFO nanoparticles inhibit ferritinophagy in granulosa cells

A-C Protein levels of GPX4 and FTL detected by Western blotting. KGN cells were treated with Cis, Cis + mPDA@DFO, or Cis + FSH-mPDA@DFO for 48 h. β-actin served as the loading control. **D-H** IF analysis and quantification of GPX4, FTL and GPX4 expression levels. Mice were administered Cis, Cis + mPDA@DFO, or Cis + FSH-mPDA@DFO for 14 days prior to analysis.

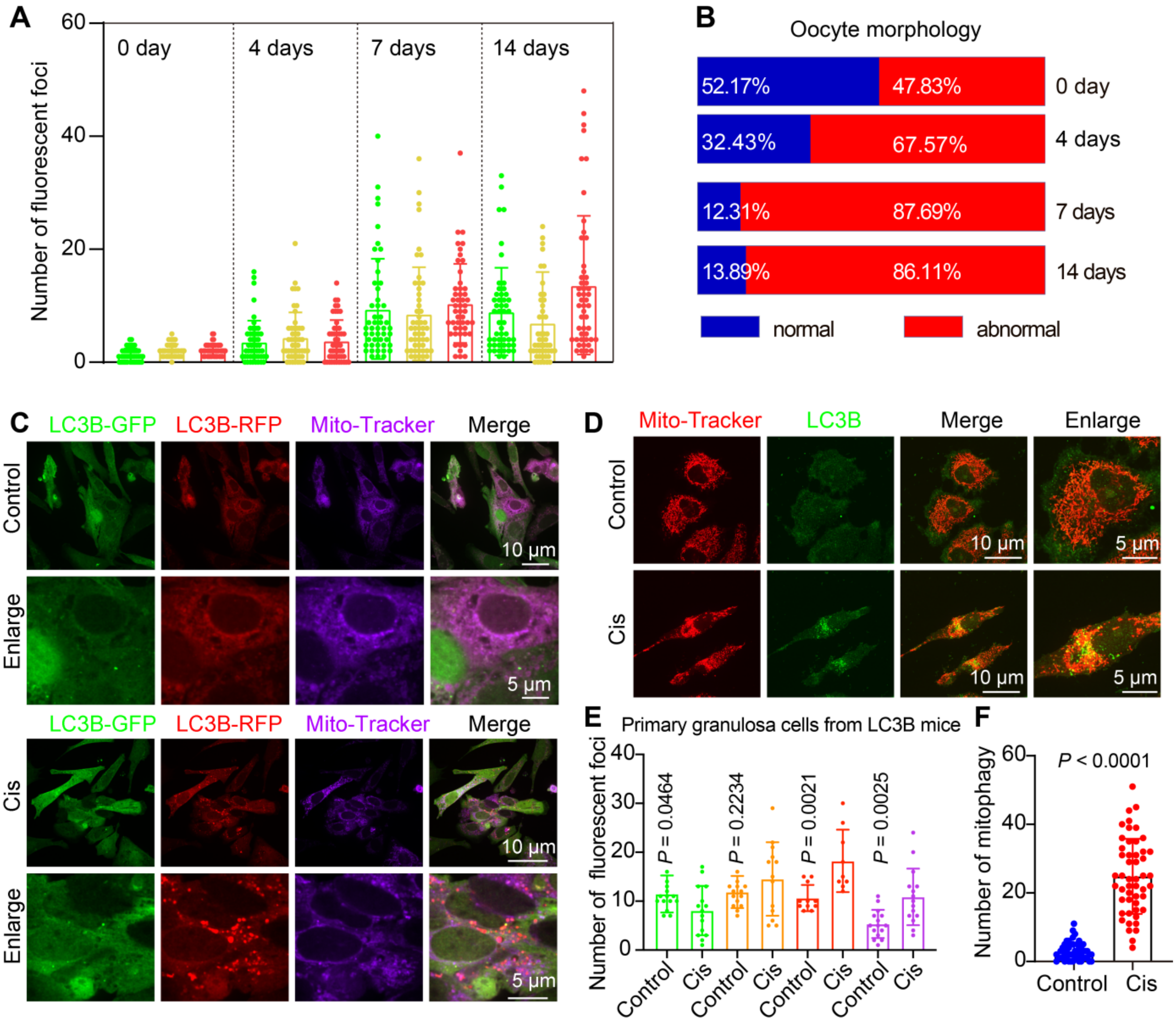

Figure S17 Cisplatin induces granulosa cells mitophagy and oocyte damage

A Number of green, yellow, and red fluorescent foci. **B** The proportions of normal and abnormal oocytes following cisplatin treatment for 4 days, 7 days, and 14 days. At least 100 oocytes were counted in each group. **C** Primary granulosa cells isolated from 8-week-old CAG-RFP-GFP-LC3B transgenic reporter mice were co-stained with Mito-Tracker. These cells were cultured with or without cisplatin for 24 hours. **D** KGN cells were co-stained with Mito-Tracker and LC3B after being cultured with or without cisplatin for 48 hours. **E** The quantification of fluorescent foci in primary granulosa cells from LC3B mice, which were treated with cisplatin for 24 hours and co-stained with Mito-Tracker and DAPI. **F** The number of double-positive foci for LC3B and Mito-Tracker, indicating mitophagy. KGN cells were cultured with cisplatin for 48 hours and co-stained with Mito-Tracker and LC3B.

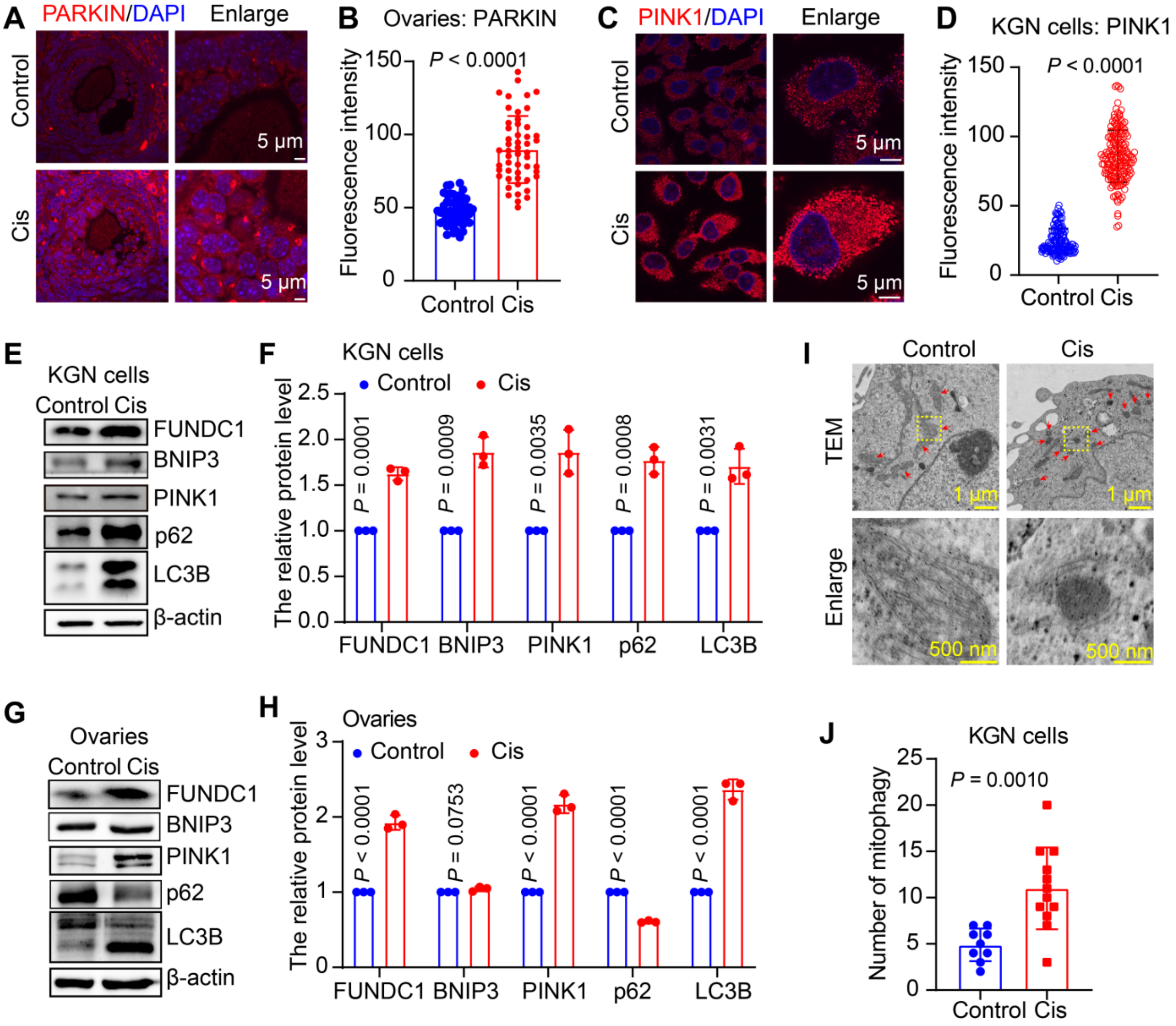

Figure S18 Cisplatin chemotherapy induces mitophagy in granulosa cells

A-B IF staining and fluorescence intensity analysis of PARKIN in ovarian sections. Mice were treated with or without cisplatin for 14 days. **C-D** IF staining and fluorescence intensity analysis of PINK1 in KGN cells, which were treated with or without cisplatin for 48 hours. **E-H** Western blotting analysis of FUNDC1, BNIP3, PINK1, p62, and LC3B in cisplatin-treated KGN cells (G) and ovaries (H). **I-J** TEM analysis of mitophagy alterations.

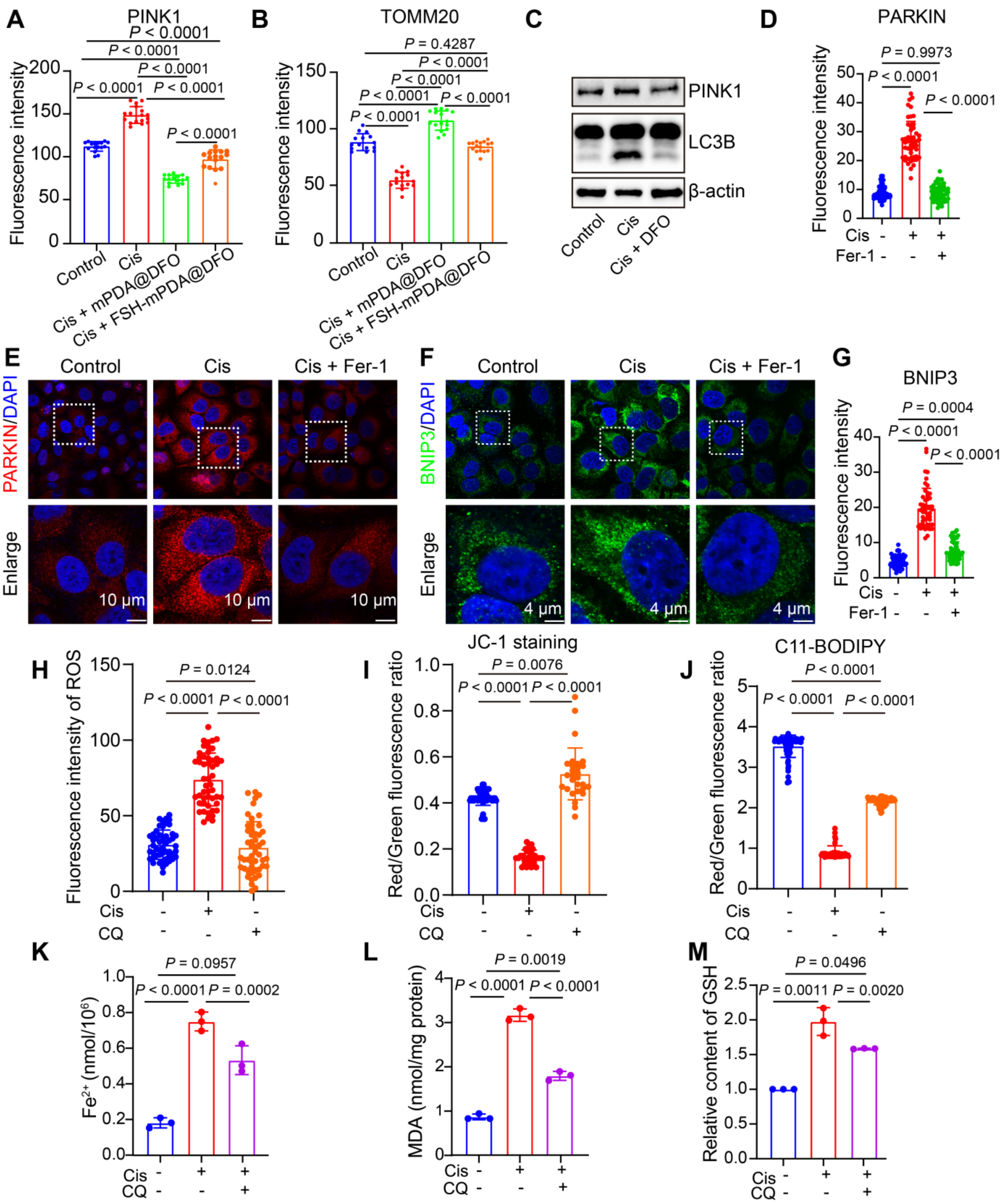

Figure S19 Autophagy and ferroptosis synergistically regulate cisplatin-induced granulosa cells damage

A-B Statistical analysis of PINK1 and TOMM20 expression in ovarian tissue sections. The mice were treated with Cis, Cis + mPDA@DFO, Cis + FSH-mPDA@DFO. **C** The protein level of PINK1 and LC3B in KGN cells treated with Cis and Cis + DFO. **D-G** IF staining and

statistical analysis of PINK1 and BNIP3 expression in KGN cells treated with Cis and Cis + DFO. **H** Intracellular ROS levels were measured using a Reactive Oxygen Species Assay Kit in KGN cells treated with Cis and Cis + CQ. **I** The decrease in mitochondrial membrane potential is indicated by a fluorescence emission shift from red (aggregates) to green (monomer) in KGN cells treated with Cis and Cis + CQ. **J** Lipid ROS levels were detected using C11-BODIPY 581/591 probes. **K-M** The levels of Fe^{2+} (K), MDA (L), and GSH (M) in the indicated group in KGN cells treated with Cis and Cis + CQ.

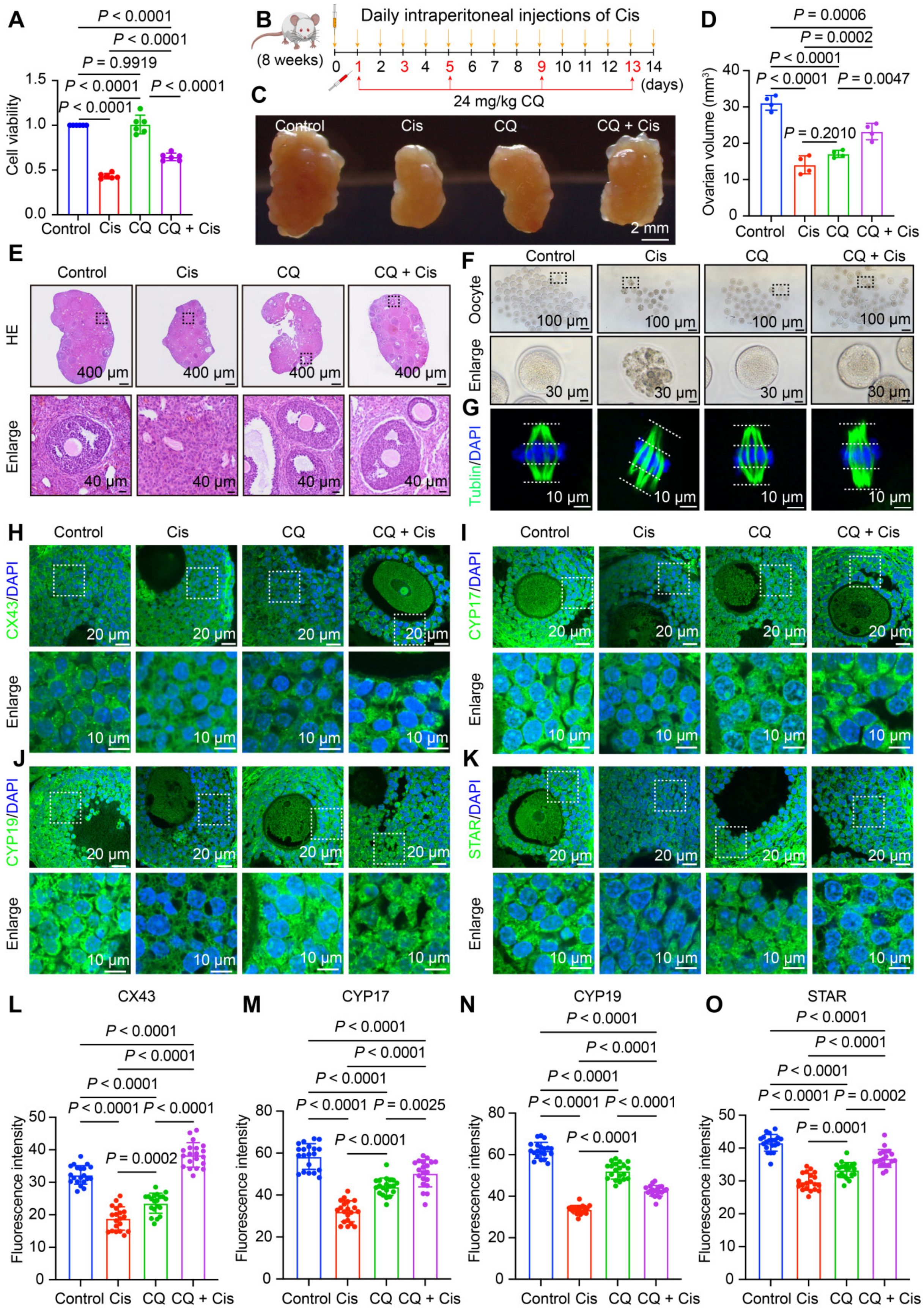

Fig. S20 Effects of chloroquine treatment on cisplatin- induced POF

A Cell viability assessed by CCK-8 assay in KGN granulosa cells treated with Cis, CQ, or CQ + Cis for 48 hours. **B** Schematic diagram of the experimental protocol for *in vivo* administration of Cis, CQ, or CQ + Cis in mice. **C-D** Representative images of ovaries and quantitative analysis of ovarian volume in different treatment groups. **E** Histological evaluation of ovarian follicles by HE staining. **F-G** Representative images of ovulated oocytes. Immunofluorescence staining of oocytes showing chromosomes (DAPI, blue) and spindle microtubules (α-Tubulin, green). **H-O** Immunofluorescence staining (H-K) and quantitative analysis (L-O) of key ovarian markers: gap junction protein Connexin 43 (CX43; H, L), and steroidogenesis enzymes CYP17 (I, M), CYP19 (J, N), and STAR (K, O).

Supporting table

Table S1 Antibodies

Antibodies	Vendors; Cat. No.	Source	Dilution/Applications
β-actin	Proteintech; 60008-1-Ig	Mouse	1: 5000 (WB)
TFRC	Beyotime Biotechnology; AF8136	Rabbit	1: 1000 (WB); 1: 200 (IF); 1: 200 (IHC)
SLC7A11	Proteintech; 26864-1-AP	Rabbit	1: 1000 (WB); 1: 400 (IHC)
GPX4	Proteintech; 67763-1-Ig	Mouse	1: 2000 (WB); 1: 1000 (IF)
TOMM20	Beyotime Biotechnology; AF1717	Rabbit	1: 1000 (WB); 1: 200 (IF)
P62	Cell Signaling Technology; #88588	Mouse	1: 1000 (WB)
FUNDC1	Proteintech; 28519-1-AP	Rabbit	1: 10000 (WB)
LC3B	Cell Signaling Technology; #83506	Mouse	1: 1000 (WB); 1: 200 (IF)
BNIP3	Proteintech; 68091-1-Ig	Rabbit	1: 10000 (WB)
FTL	Beyotime Biotechnology; AF6933	Rabbit	1: 1000 (WB)
FPN1	Proteintech; 26601-1-AP	Rabbit	1: 1000 (WB)
NCOA4	SANTA; sc-373739	Mouse	1: 500 (WB)

PARKIN	SERVICEBIO; GB114834	Rabbit	1: 200 (IF)
CYP17A1	Proteintech; 14447-1-AP	Rabbit	1: 50 (IF)
CYP19A1	Beyotime Biotechnology; AF6231	Rabbit	1: 200 (IF)
CYP11A1	PTM BIO; PTM-5370	Mouse	1: 50 (IF)
CX43	Proteintech; 26980-1-AP	Rabbit	1: 200 (IF)
RDX	Proteintech; 13790-1-AP	Rabbit	1: 50 (IF)
STAR	Proteintech; 12225-1-AP	Rabbit	1: 200 (IF)
α-Tublin	Cell Signaling Technology; #2144S	Rabbit	1: 100 (IF)
αSMA	BAIJIA; IMB0148	Mouse	1: 200 (IF)

Table S2 Primers

**Primer**	**Sequence**	**Application**
Tfrc$^{fl/fl}$-F	GAAATAGAAACCCTCGAAAGGCTG	Genetype
Tfrc$^{fl/fl}$-R	TTCTGTAAATGGTAGATGAAGGCT	Genetype
Foxl2-CreERT2-F	GTGATGAGGTTCGCAAGA	Genetype
Foxl2-CreERT2-R	CGGACCGACGATGAAG	Genetype
LC3B-F	CATGGACGAGCTGTACAAGT	Genetype
LC3B-R	CACCGTGATCAGGTACAAGGA	Genetype
p16-F-Mouse	GAACTCTTTCGGTCGTACCC	qPCR
p16-R-Mouse	CGAATCTGCACCGTAGTTGA	qPCR
p21-F-Mouse	CCTGGTGATGTCCGACCTG	qPCR
p21-R-Mouse	CCATGAGCGCATCGCAATC	qPCR
Il-1β-F-Mouse	GCCACCTTTTGACAGTGATGAG	qPCR
Il-1β-R-Mouse	ATGTGCTGCTGCGAGATTTG	qPCR
Mmp2-F-Mouse	CAAGTTCCCCGGCGATGTC	qPCR
Mmp2-R-Mouse	TTCTGGTCAAGGTCACCTGTC	qPCR
Il-6-F-Mouse	AGCCAGAGTCCTTCAGAGAGAT	qPCR

Il-6-R-Mouse	AGGAGAGCATTGGAAATTGGGG	qPCR
Tgf-β-F-Mouse	CTAATGGTGGAAACCCACAACG	qPCR
Tgf-β-R-Mouse	TATCGCCAGGAATTGTTGCTG	qPCR
Ncoa4-F-Mouse	TGTGATGACAACTGTGAGAAGGAAG	qPCR
Ncoa4-R-Mouse	AGGTTCCATAGGCATTCCATTCTTG	qPCR
Tfrc-F-Mouse	ATGCCGACAATAACATGAAGGC	qPCR
Tfrc-R-Mouse	ACACGCTTACAATAGCCCAGG	qPCR
Slc7a11-F-Mouse	GGCATACTCCAGAACACGGG	qPCR
Slc7a11-R-Mouse	CAGTTCCACCCAGACTCGAA	qPCR
Homx1-F-Mouse	AAGCCGAGAATGCTGAGTTCA	qPCR
Homx1-R-Mouse	GCCGTGTAGATATGGTACAAGGA	qPCR
Acsl4-F-Mouse	CTCACCATTATATTGCTGCCTGT	qPCR
Acsl4-R-Mouse	TCTCTTTGCCATAGCGTTTTTCT	qPCR
Cp-F-Mouse	CTTAGCCTTGGCAAGAGATAAGC	qPCR
Cp-R-Mouse	GGCCTAAAAACCCTAGCCAGG	qPCR
Gss-F-Mouse	CAAAGCAGGCCATAGACAGGG	qPCR
Gss-R-Mouse	AAAAGCGTGAATGGGGCATAC	qPCR
Pcbp1-F-Mouse	GACGCCGGTGTGACTGAAA	qPCR
Pcbp1-R-Mouse	GTCAGCGTGATGATCCTCTCC	qPCR
Map1lc3a-F-Mouse	GACCGCTGTAAGGAGGTGC	qPCR
Map1lc3a-R-Mouse	CTTGACCAACTCGCTCATGTTA	qPCR
Ftl1-F-Mouse	CCATCTGACCAACCTCCGC	qPCR
Ftl1-R-Mouse	CGCTCAAAGAGATACTCGCC	qPCR
Trf-F-Mouse	GCTGTCCCTGACAAAACGGT	qPCR
Trf-R-Mouse	CGGAAGGACGGTCTTCATGTG	qPCR
Steap-F-Mouse	AGACCTGGCACTGCTATGTC	qPCR
Steap-R-Mouse	CACTTGAGCTAAGGAGGGGAA	qPCR
Gpx4-F-Mouse	GATGGAGCCCATTCCTGAACC	qPCR

Gpx4-R-Mouse	CCCTGTACTTATCCAGGCAGA	qPCR
β-ACTIN-F-Human	CATGTACGTTGCTATCCAGGC	qPCR
β-ACTIN-R-Human	CTCCTTAATGTCACGCACGAT	qPCR
NCOA4-F-Human	ACTTTCAAGATGTAACCGTTGGG	qPCR
NCOA4-R-Human	GGCTGCTCAACTCTTGTCCA	qPCR
TFRC-F-Human	GAGGACGCGCTAGTGTTCTT	qPCR
TFRC-R-Human	GGCTGAACCGGGTATATGACA	qPCR
SLC7A11-F-Human	TTCATGTCCGCAAGCACACT	qPCR
SLC7A11-R-Human	AGCAACTGCCAGCCCAATAA	qPCR
HOMX1-F-Human	CTGCGTTCCTGCTCAACATC	qPCR
HOMX1-R-Human	GGGGCAGAATCTTGCACTTT	qPCR
ACSL4-F-Human	TTTTGCGAGCTTTCCGAGTG	qPCR
ACSL4-R-Human	ATAGCAGTACAGCCAAGGCA	qPCR
CP-F-Human	CGGCCATAGCTTCCAATACA	qPCR
CP-R-Human	GCCAGATTTGGTGTCTTCATTT	qPCR
GSS-F-Human	GCGGAGGAAAGGCGAACTA	qPCR
GSS-R-Human	AGAGCGTGAATGGGGCATAG	qPCR
PCBP1-F-Human	ATATCAACAGCTCCATGACCAACAG	qPCR
PCBP1-R-Human	CTTACACCCGCCTTTCCCAATC	qPCR
MAP1LC3A-F-Human	GCCTTCTTCCTGCTGGTGAAC	qPCR
MAP1LC3A-R-Human	AAGCCGTCCTCGTCTTTCTCC	qPCR
FTL1-F-Human	TGGAGGCAGCCGTCAACAG	qPCR
FTL1-R-Human	ACGCCTTCCAGAGCCACATC	qPCR
TRF-F-Human	ATCAGCAGAGACCACCGAAGAC	qPCR
TRF-R-Human	ACAGGCACCAGACCACACTTG	qPCR
STEAP3-F-Human	TTCGCCGCGGACCTT	qPCR

STEAP3-R-Human	TACTATCGCTGTCCACCAGG	qPCR
GPX4-F-Human	CCTTTGCCGCCTACTGAAGC	qPCR
GPX4-R-Human	GGAAAACTCGTGCATGGAGC	qPCR
